# Cellular Responses Induced by NCT-503 Treatment on Triple-Negative Breast Cancer Cell Lines: A Proteomics Approach

**DOI:** 10.3390/biomedicines12051087

**Published:** 2024-05-14

**Authors:** Ioana-Ecaterina Pralea, Radu-Cristian Moldovan, Adrian-Bogdan Țigu, Cristian-Silviu Moldovan, Eva Fischer-Fodor, Cristina-Adela Iuga

**Affiliations:** 1 Department of Proteomics and Metabolomics, Research Center for Advanced Medicine–MedFuture, “Iuliu Hațieganu” University of Medicine and Pharmacy Cluj-Napoca, Louis Pasteur Street 4-6, 400349 Cluj-Napoca, Romania; pralea.ioana@umfcluj.ro (I.-E.P.); moldovan.radu@umfcluj.ro (R.-C.M.); 2 Department of Pharmaceutical Analysis, Faculty of Pharmacy, “Iuliu Hațieganu” University of Medicine and Pharmacy, Louis Pasteur Street 6, 400349 Cluj-Napoca, Romania; 3 Department of Translational Medicine, Research Center for Advanced Medicine–MedFuture, “Iuliu Hațieganu” University of Medicine and Pharmacy Cluj-Napoca, Louis Pasteur Street 6, 400349 Cluj-Napoca, Romania; bogdan.tigu@umfcluj.ro; 4 Department of BioNanoPhysics, Research Center for Advanced Medicine–MedFuture, “Iuliu Hațieganu” University of Medicine and Pharmacy Cluj-Napoca, Louis Pasteur Street 6, 400349 Cluj-Napoca, Romania; moldovan.cristian1994@gmail.com; 5 Tumor Biology Department, Institute of Oncology “Prof. Dr. Ion Chiricuță”, 400015 Cluj-Napoca, Romania; fischer.eva@iocn.ro

**Keywords:** TNBC, PHGDH inhibitor, NCT-503, proteome analysis, GSEA, targetable proteins, mass spectrometry proteome analysis, shotgun proteomics

## Abstract

Breast cancer (BC) remains one of the leading causes of mortality among women, with triple-negative breast cancer (TNBC) standing out for its aggressive nature and limited treatment options. Metabolic reprogramming, one of cancer’s hallmarks, underscores the importance of targeting metabolic vulnerabilities for therapeutic intervention. This study aimed to investigate the impact of de novo serine biosynthetic pathway (SSP) inhibition, specifically targeting phosphoglycerate dehydrogenase (PHGDH) with NCT-503, on three TNBC cell lines: MDA-MB-231, MDA-MB-468 and Hs 578T. First, MS-based proteomics was used to confirm the distinct expression of PHGDH and other SSP enzymes using the intracellular proteome profiles of untreated cells. Furthermore, to characterize the response of the TNBC cell lines to the inhibitor, both in vitro assays and label-free, bottom-up proteomics were employed. NCT-503 exhibited significant cytotoxic effects on all three cell lines, with MDA-MB-468 being the most susceptible (IC_50_ 20.2 ± 2.8 µM), while MDA-MB-231 and Hs 578T showed higher, comparable IC_50s_. Notably, differentially expressed proteins (DEPs) induced by NCT-503 treatment were mostly cell line-specific, both in terms of the intracellular and secreted proteins. Through overrepresentation and Reactome GSEA analysis, modifications of the intracellular proteins associated with cell cycle pathways were observed in the MDA-MBs following treatment. Distinctive dysregulation of signaling pathways were seen in all TNBC cell lines, while modifications of proteins associated with the extracellular matrix organization characterizing both MDA-MB-231 and Hs 578T cell lines were highlighted through the treatment-induced modifications of the secreted proteins. Lastly, an analysis was conducted on the DEPs that exhibited greater abundance in the NCT-503 treatment groups to evaluate the potential chemo-sensitizing properties of NCT-503 and the druggability of these promising targets.

## 1. Introduction

Breast cancers account for 26.4% of all new cancer cases in European females, surpassing lung cancer cases, according to Cancer today statistics of 2022, published by the International Research Agency on Cancer [1]. BCs are characterized by a high degree of diversity; this heterogeneity is expressed both in an inter- and intra-tumor manner but also spatiotemporally, characterized by a dynamic molecular variation during progression that is also shaped by the interaction and distribution of different cell populations composing the tumor [2].

Triple-negative breast cancer represents approximately 15–20% of all BC cases. Due to high molecular heterogenicity, it is reported as the most aggressive BC subtype, being characterized by low overall survival and the highest metastases rates [3,4]. Therefore, TNBC is still an unmet clinical need that might be addressed by subtyping based on metabolic vulnerabilities, as highlighted in a recent review [5]. Several attempts to subclassify TNBC have been mostly made based on genomics and transcriptomics profiles, the most notable being those of Lehmann et al. [6,7] (four TNBC subtypes, based on gene expression profiles of TNBC samples), Beatty et al. [8] (two TNBC subtypes, based on the metabolome profile of TNBC cell lines) and, most recently, Gong et al. [9] (three metabolic-pathway-based TNBC subtypes, based on the multi-omics data of TNBC samples).

**Figure 1:**
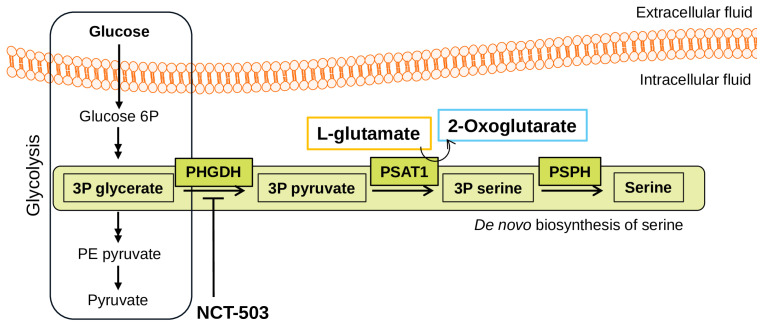
Serine biosynthesis pathway. The glycolysis pathway—as a general overview (left side)—and the de novo biosynthesis of serine is highlighted in green shades with the key enzymes: PHGDH—3-phosphoglycerate dehydrogenase; PSAT1—phosphoserine transaminase; PSPH—phosphoserine phosphatase.

Metabolic reprogramming is a hallmark of cancer that is responsible for the abnormal development of malignant cells. Cancer cells alter their macromolecule metabolism (e.g., carbohydrates, lipids, nucleic and amino acids) in order to fulfill the high requirements of energy and nutrient biosynthesis. Rapidly proliferating cells (including TNBCs) display, beside elevated glucose and glutamine consumption, a high dependency on non-glutamine amino acids, such as serine, to provide most of the needed carbon and nitrogen units [10,11]. These cells can obtain exogenous serine through dedicated transporters (such as ASCT2 [12]) but can also synthesize it from glucose through the de novo serine biosynthetic pathway (SSP). Glycolytic intermediate 3-phosphoglycerate (3PG) is the node connecting the glycolytic and SSP pathways, in which 3-phosphoglycerate dehydrogenase (PHGDH) is the first and rate-limiting enzyme, responsible for obtaining 3-phosphonooxypyruvate (Figure 1). Phosphoserine transaminase (PSAT1) and phosphoserine phosphatase (PSPH) enzymes are responsible for the sequential enzymatic reactions resulting in the synthesis of L-serine amino acid.

The SSP appears to be a central hub for sustaining the needs of malignant cells due to its role in providing building blocks for protein, lipid, and nucleotide biosynthesis [13] and replenishment of the tricarboxylic acid cycle through the production of α-ketoglutarate (2-oxoglutarate). Moreover, studies have reported the overexpression of SPP enzymes in various types of cancers (breast cancers–including TNBC—melanoma, colon cancer, glioma, cervical adenocarcinoma, lung and thyroid cancers, and leukemia) associated with the genomic amplification of PHGDH, with resistance to therapy [14,15,16] and generally a more aggressive pathology [15,16,17,18,19,20].

The role of PHGDH as a potential target reemerged following the discovery of two potent inhibitors, CBR-5884 and NCT-503 [21,22]. NCT-503, a piperazine derivative, was first reported by Pacold et al. [22] in 2016 as a promising non-competitive and reversible inhibitor of PHGDH. NCT-503 is the product of an extensive screening of the NIH Small Molecule Repository (MLSMR) library, filtering of pan-dehydrogenase inhibitors and subsequent chemistry optimization. The same study confirmed that NCT-503 treatment was effective only in PHGDH-dependent cell lines, resulting in reduced one-carbon unit incorporation into nucleotides (regardless of exogenous serine) through SHMT1 activation. An in vivo experiment presented in the same study confirmed NCT-503 presence in the tumor tissue and revealed that growth and weight reduction was only observed in PHGDH-dependent MDA-MB-468 xenografts. Most of the in vitro studies on NCT-503 inhibition effects published over last decade show a similar pattern in terms of study design, regardless of the cancer type: (i) study of SSP augmentation and (ii) use of in vitro models for NCT-503 treatment investigation as an inhibitor of the SSP rate-limiting PHGDH. Białopiotrowicz et al. [23] reported PHGDH and PSAT1 to be upregulated in Burkitt lymphoma (BL) via a MYC/ATF4-dependent mechanism, with NCT-503 treatment leading to the decreased proliferation and clonogenicity of BL cells in vitro. In other studies, PHGDH enzyme augmentation was associated with resistance to a therapeutic agent in non-small cell lung cancer [14] (NSCLC) and in hepatocellular carcinoma (HCC) [16]. In these cases, NCT-503 treatment was proven to resensitize NSCLC cells to erlotinib and act synergistically with sorafenib to suppress HCC cell growth in sorafenib-resistant MHCC97L cells. In high-risk neuroblastoma, MYCN amplification was correlated with SPP enzyme overexpression [15], with NCT-503 treatment inhibiting the growth and survival of MYCN-amplified cell lines, including doxorubicin chemo-resistant BE(2)-C and SMS-KCNR cell lines.

Understanding all the omics dimensions (e.g., the genome, transcriptome, proteome and metabolome) of cancer cell metabolic adaptations is crucial for innovation in medicine.

Considering the heterogeneity of TNBC, the aim of this study was to assess the impact of NCT-503 on a panel of TNBC cell lines using a proteomic approach. The obtained profiles of both intracellular and secreted proteins were used for metabolic pathway reconstruction in order to better understand the cellular response to treatment. Moreover, the NCT-503-induced expression of druggable targets was investigated.

## 2. Materials and Methods

### 2.1. Cell Culturing and Assays

TNBC cell lines MDA-MB-231 (HTB-26), MDA-MB-468 (HTB-132) and Hs 578T (HTB-126) were obtained from American Type Culture Collection (Manassas, VA, USA). All cells were routinely cultured in RPMI 1640 medium supplemented with 10% heat-inactivated fetal bovine serum, 1 mM sodium pyruvate, 0.1 mM non-essential amino acids and 1% penicillin–streptomycin. All cell culture reagents were purchased from Gibco (Gibco, Grand Island, NY, USA). Cell lines were routinely maintained as monolayers in 75 cm^2^ flasks (Eppendorf AG, Hamburg, Germany) and incubated at 37 °C in a humidified 5% CO_2_ atmosphere, unless otherwise stated. Each experiment was repeated three times. In all experiments, cell number and viability were determined using an automatic cell counter (NanoEnTek, Waltham, MA, USA).

#### 2.1.1. Cell Viability Testing by MTT Assay

The effect of NCT-503 on TNBC viability was investigated using 3-(4,5-dimethylthiazol-2-yl)-2,5-diphenyl-2H-tetrazolium bromide (MTT) assay. A study for the proper cell number to be seeded in experiment conditions (48 h exposure to compounds) was conducted considering confluence of the cells (min 80%) and absorbance values. Cells were grown at a density of 6.5 × 10^3^ cells/well in 96-well flat-bottom plates, with each treatment having 3 replicates/plate. After 24 h incubation, cells were treated with different concentrations of the tested compounds (0.49–250 µM) for 48 h. Cell viability was determined after incubation for 3 h with 100 μL of 1 mg/mL MTT and crystal solubilization by 150 μL DMSO. The absorbance corresponding to the viable cells was measured at 570 nm using a spectrophotometer (BMG Labtech, Ortenberg, Germany). Blank controls (medium only), and positive controls (DMSO 100%) were included on every plate. Viability rates and IC_20_ and IC_50_ values were calculated using GraphPad Prism software (Dotmatics, San Diego, CA, USA).

#### 2.1.2. Colony Formation Assay

An equal number of cells (500 cells/well) were seeded in a 6-well plate, for both the control and treatments. After 24 h, the cells were treated with NCT-503 at IC_20_ and incubated for another 48 h in CO_2_ when the culture medium was replaced with treatment-free medium. Cells were observed daily up to 14 days and when the colonies reached the proper size (more than 50 cells/colony) or when the colonies were too close to one another, the experiment was stopped and the cells were fixed with methanol (MeOH) and stained with crystal violet (SigmaAldrich, St. Louis, MO, USA) [24]. The colonies were observed using a Zeiss Axio Vert A1 inverted microscope (Zeiss, Jena, Germany), with the colony diameter being determined using ZEN software (Zeiss, Jena, Germany) by measuring the diameter of three random colonies after staining.

#### 2.1.3. Wound Healing Assay

The cells were seeded in 96-well plates with a flat bottom for both the control and treatments. A total of 2 × 10^4^ cells were added to each well and incubated overnight. After 48 h treatment, the wound was made using a pipette tip and the healing process was monitored periodically until the control’s wounds had closed [25]. The wound was examined using a Zeiss Axio Vert A1 inverted microscope (Zeiss, Jena, Germany) and the wound area was measured using ZEN software (Zeiss, Jena, Germany). Further, images were processed using Image J Software v. 1.54i (National Institute of Health, Bethesda, MD, USA).

#### 2.1.4. Morphological Analysis by Fluorescence Microscopy

Cells grown in chamber slides (5000 cells/well) were permeabilized using 0.5% TritonX and stained with a CytoPainter Mitochondrial Staining Kit–Red Fluorescence (Abcam, Cambridge, UK) for one hour, in fresh phosphate-buffered saline (PBS), at 37 °C, under 5% CO_2_. Further, cells were washed three times with PBS buffer and fixed in 4% (*w*/*v*) paraformaldehyde for 10 min at room temperature. Phalloidin–FITC (ActistainTM 488 Fluorescent Phalloidin, Cytoskeleton, Denver, CO, USA) was added and incubated at room temperature for 30 min, in darkness. The cells were washed three more times with PBS buffer and then the nuclei were stained with 100 µg/mL DAPI (Abcam, Cambridge, UK) for 30 s, followed by another step of washing with PBS [26]. The chamber slides were mounted in ProLong Gold Antifade mounting media and incubated at room temperature for 24 h, allowing the mounting media to polymerize. Cell images were obtained using an Olympus FLUOVIEW FV1200 laser scanning fluorescence confocal microscope. Image acquisition was performed using the PLAPON60xOSC2 (1.4 NA) objective. The images were obtained using sequential mode (three channels: 405 nm, 488 nm and 543 nm excitation). Other settings for the image acquisition were determined by the software FV10-ASW v.4.2, depending on the fluorescent dyes. Images were processed using Image J Software (National Institute of Health, Bethesda, MD, USA).

#### 2.1.5. Cell Culture Procedures and Protein Extraction for Mass Spectrometry Analysis

Cells were seeded at 3.0 × 10^6^ density in 150 mm diameter cell culture dishes for the intracellular proteome experiments and incubated for 24 h to allow cell attachment. After the incubation step, the medium was replaced with 25 mL complete medium containing the IC_20_ concentrations of NCT-503 or control (DMSO 0.1% was used as solvent control) for respective cell lines. In all cases, the final concentration of DMSO in medium did not exceed 0.1%. After 48 h treatment, the medium was discarded, cells were washed with PBS and trypsin-treated for cell detach from culture dish surface. The trypsin treatment was halted by adding complete media, the cells being collected by centrifugation at 1200 rpm for 5 min at room temperature and further resuspended in complete medium. The obtained cell pellet was washed 3 more times with PBS and stored at −80 °C until further processing.

The culture procedure used for the retrieval of culture media secreted proteome was the following: cells were seeded at a density of 2.0 × 10^6^ cells for MDAs and 1.5 × 10^6^ for Hs 578T in T75 cell culture flasks. After cell attachment (24 h), the medium was replaced with fresh complete medium containing treatment at IC_20_ for each cell line and each tested compound. After 48 h treatment, cells were rigorously washed with PBS and left for 18 h in medium without serum (starvation step). The supernatant (10 mL) was collected for secreted protein retrieval while the cell pellet was used for cell number and viability determination.

### 2.2. Sample Preparation for Mass Spectrometry

#### 2.2.1. Protein Extraction

The cell pellet obtained in the case of the intracellular proteome was solubilized in 8M urea/2M thioureea (UT) buffer, protein extraction was enhanced by cell lysis induced by 5× freeze–thaw cycles in liquid nitrogen and sonication treatment for 3 × 3 s, power 50%, (Bandelin Electronic GmbH, Berlin, Germany).

Culture media-secreted proteins (10 mL) were first concentrated until the volume of 100 µL using 3 kDa cutoff filters (15 mL capacity, MilliporeSigma, Burlington, MA, USA). Proteins were precipitated using MeOH/chloroform–3/1 (*v/v*), the resulting protein pellet being solubilized in Rapigest^®^ 0.1% prepared in 50 mM ammonium bicarbonate buffer (ABC) (Waters Corporation, Milford, MA, USA) and further sonicated for 3 × 3 s. at 50% amplitude (Bandelin electronic GmbH, Berlin, Germany). After centrifugation, total protein content was measured in the supernatant using a micro-Bradford assay (BioRad Laboratories, Munich, Germany), having bovine serum albumin as a standard protein.

#### 2.2.2. Protein Digestion and Peptide Purification

A sample volume corresponding to 2 µg of protein was subjected to a reduction with dithiothreitol (added to a final concentration of 5 mM, 1 h at 60 °C) and alkylation with iodoacetamide (final concentration of 40 mM, 15 min at 37 °C). Proteolytic cleavage with trypsin (MilliporeSigma, Burlington, MA, USA) was carried out overnight at 37 °C at 1:50 ratio. The digestion was stopped with 5% acetic acid and the samples were purified using ZipTip μC18 (Millipore-Sigma, Burlington, MA, USA) according to the manufacturer’s protocol. The eluted peptides were evaporated using a vacuum concentrator (Thermo Fisher Scientific, Waltham, MA, USA) and solubilized in 20 µL of 0.1% formic acid in acetonitrile/water–2/98 (*v/v*).

### 2.3. Label Free Nano-LC-IMS-MS and Data Analysis

#### 2.3.1. Data Acquisition and Primary Data Processing

Proteome profiling data acquisition was carried out by injecting 3 µL of sample (300 ng/run) onto a reversed-phase Acquity UPLC M-class Symmetry C18 trap column (180 µm × 20 mm, 5 µm particle size, Waters Corporation, Milford, MA, USA) at a flow rate of 5 µL/min in 99% solvent A (0.1%FA in water). After 2 min of loading and washing, peptides were separated on a nanoAcquity UPLC M-Class T3 reversed-phase column (75 µm × 150 mm, 1.8 µm particle size, Waters Corporation, Wexford, Ireland) and eluted at a flow rate of 0.3 µL/min using a 90 min multistep concave gradient ranging from 5 to 85% solvent B (0.1% FA in acetonitrile). The column temperature was set to 50 °C. Eluted peptides were ionized in positive mode using the nanoESI ionization source and analyzed on a SYNAPT G2-Si HDMS instrument (Waters Corporation, Wilmslow, UK) operated in ion mobility mode using a high-definition HDMSE approach for intracellular proteome and UDMSE for the culture media-secreted protein analysis. Mass reference compound (lockmass) Glu-1-fibronopeptide B (GluFib) (100 fmol/µL) was delivered by an auxiliary pump at a flow rate of 0.3 uL/min, the spectra of the doubly charged species (*m*/*z* 785.8426) being recorded every 45 s. Source settings included a capillary voltage of 2.5 kV, source temperature of 80 °C and sampling cone voltage of 30 V. The cone gas flow was set to 30.0 L/h.

Spectra were collected over the 50–2000 *m*/*z* mass range at a scan time rate of 0.5 s. Acquisition mode: positive, resolution. For HDMS^e^ mode, no collision energy was applied to the low-energy scan, whereas for the high-energy cycles, a ramp transfer energy from 19 to 45 V was used. In UDMS^e^ for high-energy cycles, a lookup table was used to optimize precursor fragmentation in the transfer cell [27]: (i) ion mobility bins 1–19: collision energy (CE) of 4 eV; (ii) ion mobility bins 20–190: CE of 17–75 eV; (iii) ion mobility bins 191–200: CE of 4 eV. A PLGS threshold inspector (V. 2.3 build 2, Waters crop.) was used to determine optimum low-energy (LE) and high-energy settings (HE) as 300 and 30 counts, respectively. Raw MS data were processed by Progenesis QIP v.4.2 (Nonlinear Dynamics, Waters Corporation) where data were lockmass-corrected post-acquisition using the doubly charged monoisotopic ion of GluFib and aligned to the most suitable reference run identified automatically by the software. Normalization was performed using the default “normalize to all proteins” option. A target–decoy UniPortKB/Swiss-Prot Human database containing 20,361 proteins (downloaded January 2022) was used for protein identification using the following parameters: trypsin as the digestion agent, maximum missed cleavages: one; fixed modification: carbamidomethylation; variable modification: methionine oxidation. The false discovery rate (FDR) was set to <1%. Ion match requirements implemented were (i) minimum one fragment ion match per peptide ion, (ii) 3 fragment ions matched per protein identification and (iii) at least one peptide match per protein identification. Peptides with a sequence length of less than five amino acids and a mass error of more than 10 ppm were removed. Relative quantitation using non-conflicting data was applied as the protein quantitation method. The reviewed list of proteins was exported for subsequent data analysis.

#### 2.3.2. Data Analysis

A post-processing step was conducted in Metaboanalyst 6.0 (accessed on 18 January 2024) after reverse sequences were removed and average of technical replicates were averaged; proteins with more than 30% missing values were removed; and remaining missing values were estimated using k-nearest neighbors based on similar features (KNN (feature-wise) option). No further filtering was applied; data were log_10_ transformed. For differential expression analysis, a two-sample *t*-test with unequal group variance was applied with a *p*-value threshold of 0.05 and fold change |FC| ≥ 1.2. The same online tool was used for volcano plot graphical representations.

Functional analysis of the proteome data was conducted using several tools and databases. SubcellulaRVis [28] was used for cellular component gene function analysis. Gene function analysis was performed with PANTHER Classification System v.17.0 [29] using PANTHER GO-Slim Biological Process, PANTHER GO-Slim Molecular Function and PANTHER Protein Class ontologies. Graphical representations of the functional analysis were obtained using GraphPad prism (v.10).

Network visualizations were obtained using Cytoscape v 3.10.1 [30]. Protein–protein interaction (PPI) networks were obtained using StringApp v. 2.0.3 plugin for Cytoscape using the full STRING network, confidence cutoff of 0.7 and no additional interactors. Enrichment analysis and visualization for the retrieved network was performed using StringEnrichment analysis, gProfiler (included in Enrichment Table core app v2.0.5) and ClueGO (v2.5.10) and CluePedia tools (v1.5.10) [31]. The roles of the significant proteins were investigated using multiple databases: GO ontologies (molecular function—MF, and biological processes—BP), Reactome, KEGG, and Wikipathways. Also, a term (pathway) was considered enriched if a minimum of 3 proteins could be assigned to the term, and had term B-H-corrected *p*-value < 0.005. Enrichment analysis was conducted also using EnrichR online tool [32]. Omics Visualizer (v1.3.1) was used to visualize and connect supplementary data onto networks (e.g., average FC, highest condition, number of genes/term).

ReactomeGSA online tool [33] was implemented for understanding the biological significance of protein sets and their expression. The PADOG option was employed using default settings for proteomics (intensity) datasets. Results were visualized using the ClueGO and Cluepedia Cytoscape apps. A pathway was considered significative if a minimum of 3 proteins could be assigned to the term and it displayed a B-H-corrected *p*-value < 0.005.

For the investigation of the druggable proteome, the Human Proteome Atlas (Version: 23.0) database was used. Moreover, network visualization of the targetable proteins and their FDA-approved drugs was obtained using CytargetLinker app. v 4.1.0 with DrugBank linkset release 4.2.

## 3. Results

### 3.1. Proteome Heterogenity of Untreated TNBC Cell Lines

The intracellular proteome profile of untreated TNBC cell lines consisted of 2256 identified proteins. Hierarchical clustering analysis performed on these data highlighted the fact that MDA-MB-231 and MDA-MB-468 are more alike while the Hs 578T cell line has a different expression pattern (Figure S1A). For differential expression analysis, ANOVA and post hoc Tuckey HSD tests (FDR-corrected *p*-value cutoff of 0.05) were applied, obtaining 1462 significantly different proteins (Table S1). As represented in Figure S1B, 264 proteins have different expressions in all the TNBC cell lines. These proteins were implicated mostly in neutrophil degranulation (R-HSA:6798695, BH-corrected *p*-value: 3.79 × 10^−6^, 27 proteins), formation of the cornified envelope (R-HSA:6809371, BH corrected *p*-value: 5.35 × 10^−5^, 10 proteins) and amino sugar and nucleotide sugar metabolism (KEGG:00520, BH corrected *p*-value: 4.57 × 10^−3^, six proteins). A total of 323 proteins were involved in the metabolism of proteins (R-HSA:392499, 4.66 × 10^−9^, 87 proteins), cellular responses to stimuli (R-HSA:8953897, 5.04 × 10^−9^, 49 proteins), vesicle-mediated transport (R-HSA:5653656, 2.90 × 10^−6^, 36 proteins), signaling by Rho GTPases (R-HSA:194315, 1.56 × 10^−4^, 32 proteins) and the cell cycle; mitotic proteins (R-HSA:69278, 2.21 × 10^−4^, 27 proteins) can distinguish the Hs 578T cell line from the MDAs, while MDA-MB-231 can be recognized from the other two cell lines by the expression levels of 162 proteins. These proteins were assigned to the VEGFA-VEGFR2 signaling pathway (WP:3888, 9.45 × 10^−6^, 19 proteins), the citric acid (TCA) cycle and respiratory electron transport (R-HSA:1428517, 8.07 × 10^−4^, 9 proteins), and cell cycle S phase and G1/S transition. ClueGO enrichment analysis conducted on proteins responsible for MDA-MB-468 cell line differentiation from Hs 578T and MDA-MB-231 emphasized their involvement in the metabolism of amino acids and derivatives (R-HSA:71291, 2.54 × 10^−5^, 19 proteins), cholesterol biosynthesis (R-HSA:191273, 7.11 × 10^−6^, 7 proteins), mRNA Splicing (R-HSA:72172, 1.15 × 10^−5^, 15 proteins) and signaling through the RHO GTPase cycle pathway (R-HSA:9012999, 4.58 × 10^−4^, 18 proteins).

Data regarding the expression levels of the proteins implicated in the SSP pathway were inferred from the proteome profile data (Figure S1C). Compared with MDA-MB-231, both the MDA-MB-468 and Hs 578T cell lines have higher expression of the target enzyme PGHDH. Higher expression levels for PSPH are characteristic of the MDA-MB-468 cell line while higher PSAT1 protein levels are found in the Hs 578T cell line.

Additionally, an overview of the proteomics information, which includes the results of functional analysis conducted on the intracellular proteome and secreted protein profiles of each TNBC cell lines, can be found in Supplementary Information S1 and Figures S2 and S3.

### 3.2. TNBC Cells Response to NCT-503 Treatment

#### 3.2.1. Results of In Vitro Assays

**Figure 2:**
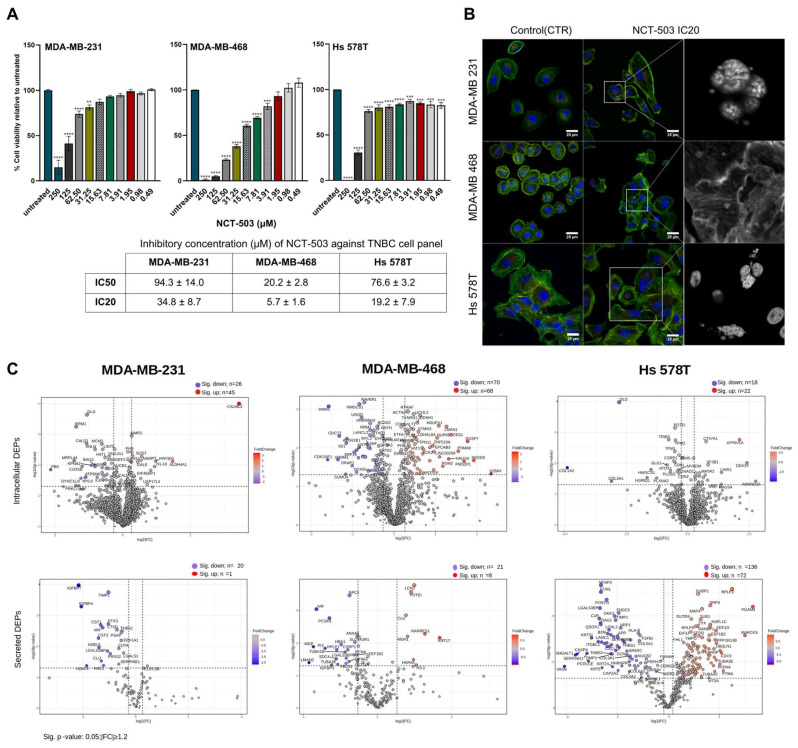
(**A**). In vitro evaluation of NCT-503 antiproliferative effect against TNBC cell panel. Cells were exposed to the indicated concentrations of NCT-503 for 48 h. Untreated cells (blank control) were exposed only to culture media while positive controls were represented by cells treated with 100% DMSO. Cell viability is expressed as percentage of control (set as 100%) and represented as mean ± SEM (n = 3). Data were analyzed by one-way ANOVA followed by Dunnett’s multiple-comparison test. The asterisks, **, *** and **** indicate significant difference at, *p* < 0.01, *p* < 0.001 and *p* < 0.0001, respectively, compared to corresponding control; (**B**) Morphologic impact of NCT-503 treatment at IC_20_ against TNBC cell panel; (**C**) Volcano plot representations of differentially expressed proteins (DEPs) induced by NCT-503 treatment in each TNBC cell line (NCT-503 vs. control).

The cytotoxicity potential of NCT-503 on the three TNBC cell lines was evaluated using the MTT assay. A 48 h exposure design was utilized, with the compound tested across a concentration range of 0.48 to 250 μM. The NCT-503 treatment presented antiproliferative effects, expressed in a dose-dependent manner on all three TNBC cell lines. The cell lines responded differently, as reflected by the IC_50_ values (Figure 2A), MDA-MB-468 proliferation being highly impaired by the NCT-503 treatment (IC_50_ value of 20.2 ± 2.8 µM). On the other hand, Hs 578T and MDA-MB-231 presented similar IC_50_ values of 93.4 ± 14.0 µM and 76.6 ± 3.2 µM, respectively.

Both the IC_20_ and IC_50_ doses were used to evaluate the morphological changes induced by the NCT-503 treatment in the TNBC cell lines (Figure 2B, Supplementary Figure S4A). For the MDA-MB-231 cell line, the NCT-503 treatment at IC_20_ (34.8 ± 8.7 µM) induced multilobulate nuclei and changes in morphology associated with a tendency to shift into round-shaped cells, indicative of acute cellular stress. The morphological evaluation emphasized a less defined cytoplasmic membrane and the irregular shape of MDA-MB-468 cell lines when treated with NCT-503 (IC_20_ = 5.7 ± 1.6 µM). Visible gaps between the cells and fragmented nuclei, indicating cell death initiation, were morphological changes expressed in the Hs 578t cell line as a response to NCT-503 (IC_20_ = 19.2 ± 7.9 µM). In the case of both MDA-MB-231 and Hs 578T, the NCT-503 IC_50_ dose caused fragmented nuclei in multiple cells. Additionally, for Hs 578T, enlarged cells were displayed in the colony and the morphological changes were associated with mitotic catastrophe. Specific to MDA-MB-468, the IC_50_ dose significantly affected overall viability, with fewer cells in the microscopical field, and the cytoskeleton, which was fragmented with less defined actin fibers.

A cell line-dependent effect of NCT-503 treatment on colony formation capability was observed. The colony formation process of the MDA-MBs was inhibited by NCT-503 treatment after 14 days of incubation (Supplementary Figure S4B). In contrast, the Hs 578t cell line did not show sensitivity to the NCT-503 treatment, with the colony formation process not being disrupted. The impact of the NCT-503 treatment (IC_20_) on the wound healing capacity of TNBC cell lines was measured for 32 h when controls completed the wound closure. The NCT-503 treatment had a significant effect on the wound healing capacity of MDA-MB-468 (starting from 24 h) compared to the respective control, while both the MDA-MB-231 and Hs 578T cell lines displayed around or more than 80% of the that of the original wound closed at 32 h (Supplementary Figure S5A).

In all the tested TNBC cell lines, the treatment induced a statistically significantly higher release of LDH in the culture media, compared to chemically lysed cells (high control) (Supplementary Figure S5B). The NCT-503-treated MDA-MB-231 and Hs 578T cells presented high LDH release in the media in a dose-dependent manner. This effect was not confirmed for MDA-MB-468.

#### 3.2.2. Differentially Expressed Proteins Induced by NCT-503 Treatment

Proteins significantly modified after NCT-503 treatment were determined using univariate analysis by comparing the protein abundance levels of treated samples to their respective controls (*p* ≤ 0.05, |FC| ≥ 1.2) (Figure 2C). 

The treatment significantly altered the abundance of proteins attributed to the intracellular proteome of the MDA-MBs (further regarded as differently expressed proteins: DEPs), with 71 proteins, mostly being upregulated, attributed to the intracellular proteome of the MDA-MB-231 cell line and 138 DEPs obtained in the case of the MDA-MB-468 cell line (Table S2). On the other hand, the intracellular proteome of the Hs 578T cell line appeared to be minimally affected by the NCT-503 treatment, as indicated by the discovery of only 40 differentially expressed proteins (DEPs) through univariate analysis (Table S2). None of the proteins significantly impacted by the NCT-503 treatment (compared to their respective control) were shared by all TNBC cell lines, corroborating the idea of a different response mechanism of the cells to the same treatment. Six DEPs were common to the MDA-MBs, all of them showing the same pattern of expression: MCM3, CAPRIN1, CDK1 and MIF were the DEPs with highest abundance in the untreated groups while PHB and GARS1 were the highest expressed in the treatment groups. Three common DEPs were shared between MDA-MB-468 and Hs 578T: HMGCS1—highest in CTRs; HAUS4—highest in NCT-504 treated groups; OSTF1—different expression in these cell lines. DLD, GMPS, DDX5 and EIF4EBP1 were the DEPs commonly reported in the MDA-MB-231 and Hs 578T cell lines. Three of them shared the same pattern of expression: DLD and DDX5 showed the significantly highest expression in the control groups, while the opposite was true for EIF4EBP1. GMPS presented different patterns of expression in these cell lines after the NCT-503 treatment.

NCT-503 had an impact only on a limited number of proteins in the secreted proteome of MDA-MBs (Table S3). Specifically, 21 secreted DEPs were identified in MDA-MB-231, while 29 were found in MDA-MB-468 in response to the treatment. Conversely, 208 DEPs were revealed in the case of the Hs 578T cell line after the NCT-503 treatment. Notably, LGALS3BP exhibited a consistent response pattern to treatment across all TNBC cell lines. Two secreted DEPs were shared by the MDA-MBs, while seven and eight secreted proteins were shared between the MDA-MB-468 and Hs 578T, and MDA-MB-231 and Hs 578T, respectively. The seven differentially expressed proteins (DEPs) found in both the MDA-MB-468 and Hs 578T cell lines exhibited contrasting group expression patterns. Conversely, in the context of shared DEPs between MDA-MB-231 and Hs 578T, EEF1A1 and LGALS1 displayed varying group expressions that were cell line-dependent, while IGFBP7, TIMP1, CTSD, THBS1, CST3, SERPINE1 had the highest abundance in the control group regardless of the cell line.

Irrespective of the cell line, based on the PPI networks obtained using the StringApp tool, all intracellular DEPs were found to have more interactions among themselves, being at least partially biologically connected as a group (Supplementary Figures S6–S8). However, the DEPs obtained from the cell culture media-secreted proteins of the MDA-MB-468 cell line seemed to be a random set of proteins not very well connected.

#### 3.2.3. Enrichment Analysis

Enrichment analysis was conducted using multiple tools in order to address the limitations of this kind of analysis (namely, how enrichment is conducted in a particular tool, and the identity, version and curated status of the databases available with the enrichment tool): ClueGO and Cluepedia, StringApp enrichment, Gprofiler and EnrichR (included in Tables S4 and S5). These enrichment analysis tools were used with several common ontologies such as the GO ontologies and ReactomeDB, the KEGG and Wikipathways databases, or particular to a single tool (EnrichR) such as the MsigDB Hallmarks database (Table S6). Enrichment analysis on individual cell lines is provided in Supplementary information S2 and Supplementary Figures S9–S11. Further, the focus was placed on depicting the proteome alterations that are both common and unique in the response of the TNBC cell line to the NCT-503 treatment.

ClueGO enrichment analysis of the TNBC intracellular DEPs showed modifications of terms associated with cellular localization regulation (nucleocytoplasmic transport: GO:0046822) and the retinoblastoma gene in the cancer pathway (WP:2446) common for the MDA-MBs. Common to MDA-MB-468 and Hs 578T was the regulation of the ATP-dependent activity ontology term (GO:0043462). DEPs associated with the regulation of protein localization to the plasma membrane (GO:1903078), Orc1 removal from chromatin (R-HSA:68949) and smooth muscle contraction (R-HSA:445355) were particular to the MDA-MB-231 cell line. Instead, the Hs 578T cell line had DEPs involved in collagen biosynthesis and modifying enzymes (R-HSA:1650814). Several pathway modifications were obtained in the MDA-MB-468 cell line after the NCT-503 treatment. Here, DEPs were associated with general metabolism pathways (glycolysis/gluconeogenesis: KEGG:00010, respiratory electron transport: R-HSA:611105, lysine degradation: KEGG:00310, UCH proteinases: R-HSA:5689603), the regulation of protein localization (protein localization to mitochondrion: GO:1903747, nucleocytoplasmic transport: GO:0046824), signal transduction pathways (RAF-independent MAPK1/3 activation: R-HSA:112409 and retrograde neurotrophins signaling: R-HSA:177504), the cell cycle mitotic M-phase (Condensation of Prometaphase Chromosomes: R-HSA:2514853 and Nuclear Envelope (NE) Reassembly: R-HSA:2995410) and also autophagy (Selective autophagy: R-HSA:9663891).

Biological process GO terms associated with the regulation of peptidase activity (GO:0061134, GO:0010466) and intrinsic apoptotic signaling (GO:2001242) were commonly found in the secreted DEPs of all TNBC cell lines. Interestingly, reactome pathways associated with hemostasis (R-HSA:109582, R-HSA:114608, R-HSA:76002, R-HSA:76005) and neutrophil degranulation (R-HSA:6798695) were also found to be modified after treatment in all cell lines investigated. The MDA-MB-231 and Hs 578T cell lines shared DEPs associated with signaling by the Rho GTPases pathway (RHO GTPase Effectors: R-HSA:195258 and RHO GTPases activate PKNs: R-HSA:5625740), extracellular matrix organization (degradation of the extracellular matrix: R-HSA:1474228, GO:0030198) and metabolism of proteins (in particular, amyloid fiber formation: R-HSA:977225; regulation of insulin-like growth factor (IGF) transport and uptake by insulin-like growth factor-binding proteins (IGFBPs): R-HSA:381426 and post-translational protein phosphorylation: R-HSA:8957275). Instead, both MDA-MB-468 and Hs 578T had DEPs linked with membrane trafficking (ER to Golgi anterograde transport: R-HSA:199977; vesicle-mediated transport: R-HSA:5653656; transport to the Golgi and subsequent modification: R-HSA:948021), the Parkin–ubiquitin proteasomal system pathway (WP:2359) and nervous system development (R-HSA:9675108, R-HSA:422475). Several terms were exclusively enriched only in the Hs 578T cell line, e.g., DEPs associated with interleukin signaling (Interleukin-4 and Interleukin-13 signaling: R-HSA:6785807, gene and protein expression by JAK-STAT signaling after Interleukin-12 stimulation: R-HSA:8950505, nucleotide-binding domain, leucine rich repeat containing receptor (NLR) signaling pathways: R-HSA:168643) and signal transduction pathways (MET activates PTK2 signaling: R-HSA:8874081, RHOBTB2 GTPase cycle: R-HSA:9013418).

#### 3.2.4. Reactome GSEA Analysis

As seen previously through ClueGO analysis, intracellular proteome modifications to treatment tend to be cell line-specific. In the context of general metabolism (Figures S12–S15, Tables S7–S12), the MDA-MB-231 cell line undergoes alterations in its amino acids metabolism when subjected to NCT-503 treatment. Specifically, the metabolism of aspartate and asparagine, as well as the degradation of glycine, were upregulated as response. Moreover, the catabolism of lysine and branched-chain amino acids was downregulated. This response was also exhibited by the Hs 578T cell line. In addition, MDA-MB-231 displayed downregulation of nucleotides and steroid hormone metabolism and upregulation of the pyruvate dehydrogenase (PDH) complex. Instead, the MDA-MB-468 cell line displayed a notable decrease in the sphingolipid metabolism and increase in protein abundance involved in the mitochondrial complex I biogenesis. Hs 578T responded to treatment by downregulating the TCA cycle, metabolism of steroids and synthesis of phosphatidylinositol phosphate at the plasma membrane.

Following treatment, notable alterations in cell cycle-related pathways were observed in the MDA-MBs. Specifically, cell cycle checkpoints and associated pathways in MDA-MB-231 were suppressed, including pathways related to the mitotic G1 phase and G1/S transition. Conversely, pathways associated with the M-phase and G2/M transition were affected in MDA-MB-468 treated with NCT-503. No changes in cell cycle-associated pathways were observed in the Hs 578T cell line.

The MDA-MB-231 cell line exhibited downregulation of signaling by the GPCR pathways and upregulation of signaling by retinoic acid. Distinctive to MDA-MB-468 was the downregulation of signaling by NTRKs and FGFR and upregulation of signaling by EGFR, VEGFR2-mediated cell proliferation and RAF-independent MAPK1/3 activation. Instead, the Hs 578T cell line presented characteristic suppression of signaling by TGFB family members, ERBB4 and type 1 insulin-like growth factor 1 receptor (IGF1R).

Several modifications were highlighted by Reactome GSEA analysis in the secreted proteins of the TNBC cell panel as a response to treatment (Figures S16–S19). Both MDA-MBs exhibited downregulations of cellular stress response pathways, with MDA-MB-231 showing a specific cellular senescence response and MDA-MB-468 displaying suppression of chemical stress response pathways. Modifications of pathways associated with small-molecule transport were observed exclusively in MDA-MB-468 characterized by a decrease in plasma lipoprotein clearance and an increase in iron uptake. Instead, modifications of proteins associated with the extracellular matrix organization characterized both the MDA-MB-231 and Hs 578T cell lines’ response to treatment. A decrease in collagen and in the general extracellular matrix was seen in MDA-MB-231, while the opposite was noted in the Hs 578T cell line. In addition, an increase in elastic fiber formation was also seen in the latter cell line. A cell line-specific decrease in post-translational protein modifications was emphasized in the secreted proteins as a response to treatment. The MDA-MB-231 cell line exhibited a reduction in post-translational protein phosphorylation, whereas protein ubiquitination-related pathways were diminished in response to the NCT-503 treatment in the MDA-MB-468 cell line. However, the O-linked glycosylation pathway decreased in the Hs 578T cell line. Also, the modification of proteins related to cytokine signaling in the immune system were particularly seen in the MDA-MB-468 and Hs 578T cell lines. Both the MDA-MB-231 and Hs 578T cell lines exhibited reductions in signal transduction pathways. The MDA-MB-231 cell line demonstrated downregulation of signaling through nuclear receptors, whereas the Hs 578T cell line showed a decrease in proteins implicated in specific signaling through MET-related pathways.

### 3.3. Targetable Proteins Induced by NCT-503 Treatment

**Figure 3:**
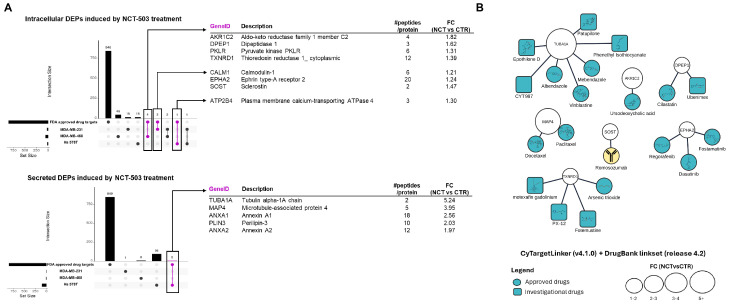
Possible drug targets induced by NCT-503 treatment. (**A**). Upset plots of upregulated TNBC targets upon NCT-503 and FDA-approved protein targets: up-intracellular upregulated DEPs; down: secreted upregulated DEPS and table description of hits. (**B**) Network representation of known FDA-approved drugs targeting proteins upregulated by NCT-503 treatment.

DEPs induced by the NCT treatment were searched against the HPA-targetable protein list. A total of 13 protein hits (Figure 3A) were obtained, 8 of them provided by the intracellular proteome protocol. The intracellular MDA-MB-468 proteome provided most of the druggable protein hits: three of them (AKR1C2, DPEP1 and TXNRD1) had FDA-approved drugs assigned (Figure 3B). The MDA-MB-231 intracellular proteome had three protein hits while two of them had market-approved antagonists (EPHA2 and SOST). Only one druggable target was assigned to the intracellular proteome of Hs 578T. In contrast, all secreted protein hits (five: TUBA1A, MAP4, ANXA1, PLIN3 and ANXA2) were provided by this cell line. From this list, only the TUBA1A protein target presented approved drugs with inhibitory properties. Table S13 includes 30 potential drug targets along with relevant data regarding their involvement in disease and function. Of these, Cathepsin B (CTSB), Protein disulfide–isomerase (P4HB) and Cystatin-B (CSTB) were found upregulated in myeloma, lung cancer or colorectal cancer.

## 4. Discussion

Characterized by the lack of estrogen, progesterone and human epidermal growth factor receptors, TNBC is a distinct form of BC characterized by a poor prognosis, high metastases rates and limited treatment options due to its heterogeneity.

As the main aim of this study was to assess the SPP inhibition impact in TNBC, the selection of TNBC-representative cell lines was made based on their dependency of PHGDH, as highlighted by Pacold et al. [22]. Cell line PHGDH dependency status was first assessed using the HPA database: the MDA-MB-231 cell line was assigned as PHGDH-independent while the MDA-MB-468 and Hs 578T cell lines were appointed to the PHGDH-dependent category, which could be confirmed by the PHGDH expression of the untreated cells (Figure S1C).

Several attempts have been made to address the high variability of TNBC through subtyping that may lead to a better understanding of the disease and facilitate a systematic and efficient therapeutic approach (Lehmann et al. [6,7] and Gong et al. [9]). Lehmann et al. [6,7] used gene profiling data of 587 TNBC cases for the original classification proposed and assigned in vitro cell line models to the subtypes. Considering this classification, two of the cell lines used in this study were designated as mesenchymal stem–like (MSL) (MDA-MB-231 and Hs 578T) while MDA-MB-468 was included in the basal-like BL1 group. The main characteristics of the MSL group, as reported by Lehmann et al. [6,7], were the enrichment of cell motility (Rho pathway), differentiation, and growth pathways (ALK pathway, TGF-β signaling and Wnt/β–catenin pathway). In addition, unique to the MSL group were the growth factor signaling pathways (including inositol phosphate metabolism, EGFR, PDGF, calcium signaling, G-protein coupled receptor ERK1/2 signaling and ABC transporter and adipocytokine signaling) and enriched genes involved in angiogenesis (including VEGFR2) and stem cells. Also, they seem to be mostly claudin-low-expressing (claudins 3, 4, and 7) but high expressing of epithelial–mesenchymal transition genes. The BL1 subtype was reported to be enriched in cell cycle and cell division pathways, increased proliferation and cell-cycle checkpoint loss, and elevated DNA damage response. High Ki-67 mRNA expression (MKI67) and nuclear IHC Ki-67 staining was particular to this group. Most recently, Gong et al. [9] applied a multi-omics approach (transcriptomic, genomic and metabolic landscape) to several cohorts of breast cancer patients for their TNBC sub-classification proposal. The authors included Hs 578T cell line into the MPS2 category while the other two cell lines were attributed to the MPS3 group (MDAMB-231 and MDA-MB-468). The MPS2 group was characterized by high chromosomal instability and copy-number alterations, with an impact especially upon glycolysis genes: this subtype is characterized by an upregulation of glycolysis, nucleotide metabolism and lactate production (including upregulation of the citric acid cycle, purine metabolism, and pyrimidine metabolism). The MPS3 was designated to the mixed subtype with partial pathway dysregulation. Hierarchical cluster analysis applied to the proteome profile of the untreated cells showed that MDA-MB-231 and MDA-MB-468 cell lines were more similar while Hs 578T displayed a different proteomic fingerprint (Figure S1A). Moreover, through statistical analysis, proteins important in the differentiation of cell lines and their implication in biological pathways were determined (Figure S1B, Table S1). Proteins differentiating Hs 578T from the other two cell lines were mostly found to be implicated in vesicle-mediated transport and membrane trafficking pathways, the metabolism of proteins, the cellular response to stimuli and cell cycle M-Phase pathways. Signaling through the Rho GTPase pathway and EPH-Ephrin signaling terms were found to be enriched in the list of proteins differentiating both the Hs 578T and MDA-MB-468 cell lines from the other two respective cell lines included in the study. Both terms are associated with cell migration, motility, cell–cell and cell–microenvironment communications, and tumor angiogenesis [34,35]. MDA-MB-468 had enriched differentiating proteins involved in the metabolism of amino acids and cholesterol biosynthesis. Instead, MDA-MB-231-differentiating proteins were mostly associated with the VEGFA-VEGFR2 signaling pathway, TCA pathway and cell cycle S-phase associated pathways. Cell viability was impaired by NCT-503 in a dose-dependent manner (Figure 2A). The inhibitory effect of the compound tested was most pronounced in the MDA-MB-468 cell line, MDA-MB-231 and Hs 578T being less sensitive to the drug.

Data-independent acquisition (DIA) combined with label-free-based quantification was employed for proteome analysis. The protocols implemented for both intracellular and secreted protein matrix retrieval and MS analysis had a similar performance regardless of the cell line, considering the cellular component, molecular function, and biological process functional analysis (Figures S2 and S3). Differential expression analysis (control vs. NCT-503 treatment) was applied for each cell line (Figure 2C, Tables S2 and S3). The DEPs found for each cell line were different in their number, identity and metabolic implications, as highlighted by enrichment analysis. After treatment, the MDA-MB-468 cell line had highest number of intracellular DEPs (138) associated with the cell cycle and the glucose, pentose phosphate and pyruvate metabolic pathways. In contrast, Hs 578T, although found to be PHGDH-dependent, presented only 40 intracellular DEPs associated mostly with collagen formation. MDA-MB-231 intracellular DEPs were found to be mostly involved in cellular senescence, protein kinase activity and apoptotic signaling. On the contrary, most secreted treatment-induced DEPs were found in the Hs 578T cell line (208) while the MDAs cell lines displayed similar number of DEPs. The Hs 578T DEPs were found to be involved in extracellular matrix organization, cytoskeleton differentiation, cell motility and adhesion, cellular response to stimuli and signal transduction pathways. DEPs associated with platelet degranulation and response to elevated platelet cytosolic calcium terms were found to be enriched in both MDAs. Platelets are associated with pathophysiologic outcomes associated with inflammation and cancer progression [36], promoting tumor cell proliferation, angiogenesis, and metastatic dissemination. Tumor cells are known to induce platelet activation and aggregation through cell surface molecules and secreted ones, or indirectly through the activation of coagulation [37]. If platelet-related pathways were mostly seen in MDA-MB-231, in MDA-MB-468, DEPs were also found to be implicated in the Parkin–ubiquitin proteasomal system pathway and associated with the antiviral mechanism by IFN-stimulated genes.

To improve the metabolic snapshot of the cellular response to NCT-503 obtained previously, GSEA analysis was conducted using the whole proteome profile obtained by each of the TNBC cell lines (Tables S7–S12). The outcome of this analysis seemed to be heterogenous and cell line-dependent, with different pathways being stimulated or inhibited as a response to treatment (Figures S12–S19). In the case of MDA-MB-231, the downregulation of the cell cycle was pronounced, with a highlight on the G0 and early G1 phase along with the downregulation of cell cycle checkpoints marking the G2/M checkpoint. Furthermore, the postmitotic pore complex reformation was downregulated, disturbing the reorganization of the cells after cell division. The interaction with extracellular matrix was significantly downregulated, the same as the metabolism of glucagon and nucleotides, while a significant upregulation was observed in aspartate metabolism and mitochondrial protein import. On the other hand, the RTK pathways, mostly modulated by FGFR, were downregulated, leading to less intense signaling via PI3K, which may have resulted in decreased cell proliferation, which can be confirmed by the microcopy analysis (Figure 2A and Figure S4A). This highlights cells with multilobulate nuclei and cells that shift from their normal shape to a round shape, indicating acute cellular stress induced by the treatment. Different situations were displayed by the MDA-MB-468 cell line, where FLT3 and Toll-like receptors cascades were upregulated. Significant upregulation was observed in EGFR, VEGFR2, FGFR 3 and FGRF 4 signaling, while in the case of FGFR 1 and FGFR 2, the signaling was downregulated. The sphingolipid metabolism, glycosphingolipid metabolism and posttranslational protein modification were significantly lowered while endocytosis mediated by clathrin was stimulated, together with the postmitotic nuclear pore complex reformation mechanism and mitochondrial tRNA aminoacylation. One similarity of the NCT-503 effect on MDA-MB-468 and MDA-MB-231 was related to the downregulated cell cycle, with the slight difference that MDA-MB-468 had a downregulated M phase and all its related bioprocesses (such as centrosome maturation and the G2/M transition). These aspects are sustained by the morphological aspects evaluated by confocal microscopy, where the cells had irregular shapes, a less defined cytoplasmic membrane and a fragmented cytoskeleton. Moreover, cell migration was significantly inhibited, and the membrane integrity was affected by the increased LDH release. MDA-MB-468 was included in the Basal-like 1 subtype and, from a molecular perspective, these TNBC cells are characterized by a high expression of cell cycle-related genes, DNA damage response genes and chemotherapy sensitivity [38,39,40,41]. Following the NCT-503 treatment, the cell cycle and signaling through RTKs were downregulated, indicating that the treatment induces cell cycle arrest and inhibits proliferation and differentiation.

Hs 578T had a different response to NCT-503 treatment compared to the other two cell lines. It seemed to be the less affected cell line; however, the overall signaling through RTKs was downregulated, highlighted in the ERBB4- and IRS-mediated signaling. Furthermore, the TGF-beta signaling was inhibited by the NCT treatment, while various effects could be observed on the metabolism, with a downregulated steroid metabolism, TCA cycle, insulin secretion and o-linked glycosylation. An increased degradation of the extracellular matrix and an intensified extracellular matrix organization was observed, along with an upregulated endosomal/vacuolar pathway. Hs 578T was included in the MPS2 category according to the classification of Gong et al. [9], being characterized by an upregulated TCA cycle, upregulated LDH, glycolysis and nucleotide metabolism. In this regard, the GSEA results showed an inhibitory effect of the NCT-503 treatment on the TCA cycle. The results were also sustained by morphological evaluations of the Hs 578T cells after the NCT-503 treatment: the cells had fragmented nuclei and significant morphological changes. One specific aspect of these Hs 578T cells treated with NCT-503 is depicted Figure S4A, where gigantic cells were displayed in the colony, indicating a potential mitotic catastrophe and an initiated programed cell death. However, the NCT-503 treatment was not effective in inhibiting migration and colony formation, indicating that Hs 578T cells were less sensitive to the treatment, or may activate compensatory mechanisms to overcome the NCT-503 treatment.

In the case of mesenchymal subtypes, the molecular characteristics highlighted were increased motility and differentiation and elevated STAT expression. Furthermore, the interaction with the extracellular matrix was affected and the epithelial-to-mesenchymal transition (EMT) was stimulated [42]. Moreover, for the EMT suppression, PI3K/mTOR and Src inhibition provided a positive outcome [43]. The profile of intracellular and secreted proteins highlighted the fact that NCT-503 treatment induced intracellular rearrangements, with changes in the interaction with the extracellular matrix and downregulation of signaling via RTKs. In the case of MDA-MB-231, migration and colony formation were inhibited, compared to the more aggressive phenotype of Hs 578T (Figures S4 and S5).

In the context of TNBC-targeting therapies, there is still a high need for new or repurposed therapeutical agents. Several studies have proved that NCT-503 can enhance the sensitivity of cells to chemotherapy, making them more susceptible to the effects of anticancer drugs [14,15,16].

Here, a set of targetable proteins and their associated FDA-approved drugs were determined using the TNBC panel proteome profile obtained after NCT-503 treatment, using the Human Proteome Atlas (HPA) (www.proteinatlas.org/humanproteome/tissue/drugg-able) [accessed on: 5 February 2024] and DrugBank databases [44], which may provide an argument to use NCT-503 as a chemo-sensitizing agent (Figure 3). According to the HPA database, there are 854 protein drug targets that have FDA-approved drugs. Most of these protein targets were predicted to be membrane-bound or secreted and seem to be involved in signal transduction pathways. The NCT-503-treatment-induced DEPs (compared to untreated cell lines) were tested against HPA database data regarding the druggable proteome. Figure 3A and Table S13 contain a list of the proteins hits obtained together with the relevant results derived from the current study, HPA database information and known FDA-approved drugs (according to the Drugbank database: www.drugbank.ca) [accessed on: 5 February 2024]. Additionally, to the list of druggable proteins, potential drug target hits were included (Table S13). Most of the protein hits could be identified in blood samples according to the HPA database; two of them, namely DPEP1 and SOST, were classified by HPA as upregulated in BC; and five of them were found to be upregulated in leukemia and myeloma: SOST, TXNRD1, PKLR, EPHA2, PLIN3.

Network visualization of the targetable proteins and their FDA-approved drugs (provided in Figure 3B) emphasizes that most drug hits are small molecules with an FDA-approved status (12) classified to the ATC L01 antineoplastic agent class (7): (i) three of these have indications of use in BC (vinblastine—ATC L01CA01, paclitaxel—ATC L01CD01 and docetaxel—ATC L01CD02). (ii) Dasatinib (ATC L01EA02) and arsenic trioxide (ATC L01XX27) are currently used in lymphoblastic and chronic myeloid leukemia and refractory or relapsed acute promyelocytic leukemia, respectively. Moreover, there is solid evidence of dasatinib being used for BC treatment (including TNBC, ER-negative BC, and ER+/PR+/HER2 + BC or metastatic BC), including several clinical studies [45,46,47]. A pertinent review regarding arsenic’s anticancer effects in BC based on in vitro study data was published in 2022 by Skoczynska et al. [48]. (iii) Fotemustine (ATC L01AD05) is used as an alkylating agent in metastatic melanoma treatment; (iv) while regorafenib (ATC L01EX05) is approved for metastatic colorectal cancer, metastatic gastrointestinal stromal tumors, and hepatocellular carcinoma. The scientific literature provides studies of regorafenib treatment impact in BC [49,50]. Other hits included two anthelmintics (ATCP 02), namely, albendazole (ATC: P02CA03) and mebendazole (ATC: P02CA51), with consistent evidence of their effect in breast and colon cancer via a mechanism of classical apoptosis and cell cycle arrest [51,52]; fostamatinib (ATC B02BX09); ursodeoxycholic acid (ATC A05AA02)—derivatives of ursodeoxycholic acid that were proven to be pro-apoptotic in BC cell lines [53]; cilastatin (ATC J01DH51) and a monoclonal antibody used to treat osteoporosis in postmenopausal women at high risk of fracture (romosozumab—ATC M05BX06). Hesse et al. [54] studied the association of romosozumab treatment in the context of breast cancer-induced bone metastases and muscle weakness.

Nevertheless, investigational drugs were also attributed to the protein targets: Epothilone D (DB01873), Patupilone (DB03010) and CYT997 (DB05147) were investigated in multiple malignances including BC, according to the DrugBank database. Ubenimex (DB03424) is currently used as adjuvant therapy in myelonous leukemia, lung cancer and nasopharyngeal cancer, with no solid evidence of use in BC; motexafin gadolinium (DB05428) is used as X-ray radiation sensitizer in relation to brain metastases resulting from non-small-cell lung cancer [55]. The closely related motexafin lutetium (DB05296) is approved by the FDA for the photodynamic treatment of BC and malignant melanomas [56]. Yang et al. [57], in an investigation into ferroptosis heterogeneity in TNBC, used PX-12 (DB05448) as a ferroptosis inducer. Phenethyl isothiocyanate (DB12695), although clinically studied in prevention and the treatment of leukemia, lung cancer, tobacco use and lymphoproliferative disorders, was shown to hamper the growth and progression of in vitro HER2-positive breast and ovarian carcinoma [58].

## 5. Conclusions

The de novo serine biosynthesis pathway is considered one of the metabolic junctions involved in TNBC pathogenesis. This study provided an insight into the intracellular and secreted proteins of three TNBC cell lines (MDA-MB-231, MDA-MB-468 and Hs 578T) which express different levels of PHGDH, along with protein changes that appear as response to NCT-503 treatment (a PHGDH inhibitor).

A higher protein expression similarity level was observed between the MDA-MB-231 and MDA-MB-468 cell lines, compared to Hs 578T, results which are in accordance with previous classifications based on multi-omics approaches. PHGDH expression among these three cell lines was highest in MDA-MB-468, which makes it more susceptible to NCT-503 treatment. Still, the impact of NCT-503 was highly heterogeneous between the three studied cell lines, mainly impacting the cell cycle, the cell–extracellular matrix interactions and several metabolic pathways.

Additionally, the potential use of NCT-503 as a chemosensitizer in TNBC was investigated by identifying possible drug targets enhanced by the treatment, which revealed a set of 11 targetable proteins along with their therapeutically approved drugs.

## Data Availability

Data are contained within the article.

## Supplementary Materials

The following supporting information can be downloaded at: https://www.mdpi.com/article/10.3390/biomedicines12051087/s1: Supplementary information S1: Overview of the intracellular and secreted proteome profile analysis; Supplementary information S2: TNBC cell line response to NCT-503 treatment; Figure S1. Heterogeneity of untreated TNBC cell line through proteome profiling; Figure S2. Overview of intracellular proteome profiles; Figure S3. Overview of the secreted protein profiles; Figure S4. NCT-503 treatment impact on cellular properties of TNBC cell panel; Figure S5. In vitro assays of NCT-503 treatment impact on TNBc cell lines; Figure S6. Protein–protein interaction network of DEPs induced by NCT-503 treatment in MDA-MB-231 cell line; Figure S7. Protein–protein interaction network of DEPs induced by NCT-503 treatment in MDA-MB-468 cell line; Figure S8. Protein–protein interaction network of DEPs induced by NCT-503 treatment in Hs 578T cell line; Figure S9. Enrichment analysis of MDA-MB-231 DEPs using ClueGO Enrichment tool; Figure S10. Enrichment analysis of MDA-MB-468 DEPs using ClueGO Enrichment tool; Figure S11. Enrichment analysis of Hs 578T DEPs using ClueGO Enrichment tool; Figure S12. Network representation of Reactome GSEA pathways for the intracellular proteome of TNBC cell lines treated with NCT-503; Figure S13. Network representation of ReactomeGSEA analysis performed on the intracellular protein profiles of NCT-503 treatment and control group of MDA-MB-231; Figure S14. Network representation of ReactomeGSEA analysis performed on the intracellular protein profiles of NCT-503 treatment and control group of MDA-MB-468; Figure S15. Network representation of ReactomeGSEA analysis performed on the intracellular protein profiles of NCT-503 treatment and control group of Hs 578T; Figure S16. Network representation of Reactome GSEA pathways for the secreted proteome of TNBC cell lines treated with NCT-503; Figure S17. Network representation of Reactome GSEA analysis performed on the secreted protein profiles of NCT-503 treatment and control group of MDA-MB-231; Figure S18. Network representation of Reactome GSEA analysis performed on the secreted protein profiles of NCT-503 treatment and control group of MDA-MB-468; Figure S19. Network representation of Reactome GSEA analysis performed on the secreted protein profiles of NCT-503 treatment and control group of Hs 578T; Table S1. Significantly differently expressed proteins in untreated TNBC cell panel; Table S2. Intracellular DEPs after NCT-503 treatment at IC20; Table S3. Secreted differentially expressed proteins (DEPs) after NCT-503 treatment at IC20; Table S4. ClueGO Enrichment analysis results of identified DEPs after NCT-503 treatment; Table S5. Gprofiler and String Enrichment analysis results of identified DEPs after NCT-503 treatment; Table S6 EnrichR Enrichment analysis results of identified DEPs after NCT-503 treatment; Table S7 Reactome GSEA analysis results of intracellular proteome profiles of NCT-503 vs. CTR groups of MDAMB231 cell line; Table S8. Reactome GSEA analysis results of secreted proteome profiles of NCT-503 vs. CTR groups of MDAMB231 cell line; Table S9. Reactome GSEA analysis results of intracellular proteome profiles of NCT-503 vs. CTR groups of MDAMB468 cell line; Table S10. Reactome GSEA analysis results of secreted proteome profiles of NCT-503 vs. CTR groups of MDAMB468 cell line; Table S11. Reactome GSEA analysis results of intracellular proteome profiles of NCT-503 vs. CTR groups of Hs 578T cell line; Table S12. Reactome GSEA analysis results of secreted proteome profiles of NCT-503 vs. CTR groups of Hs 578T cell line; Table S13. Druggable proteins induced by NCT-503 treatment in TNBC cell lines.
