# Supplementary material for: Cellular Responses Induced by NCT-503 Treatment on Triple-Negative Breast Cancer Cell Lines: A Proteomics Approach
Source: Biomedicines. 2024 May 14;12(5):1087. doi: 10.3390/biomedicines12051087 (PMC11117597; doi:10.3390/biomedicines12051087)
Supplement: Supplementary file 1 [file biomedicines-12-01087-s001.zip › Supplementary figures S1-S19.pdf]

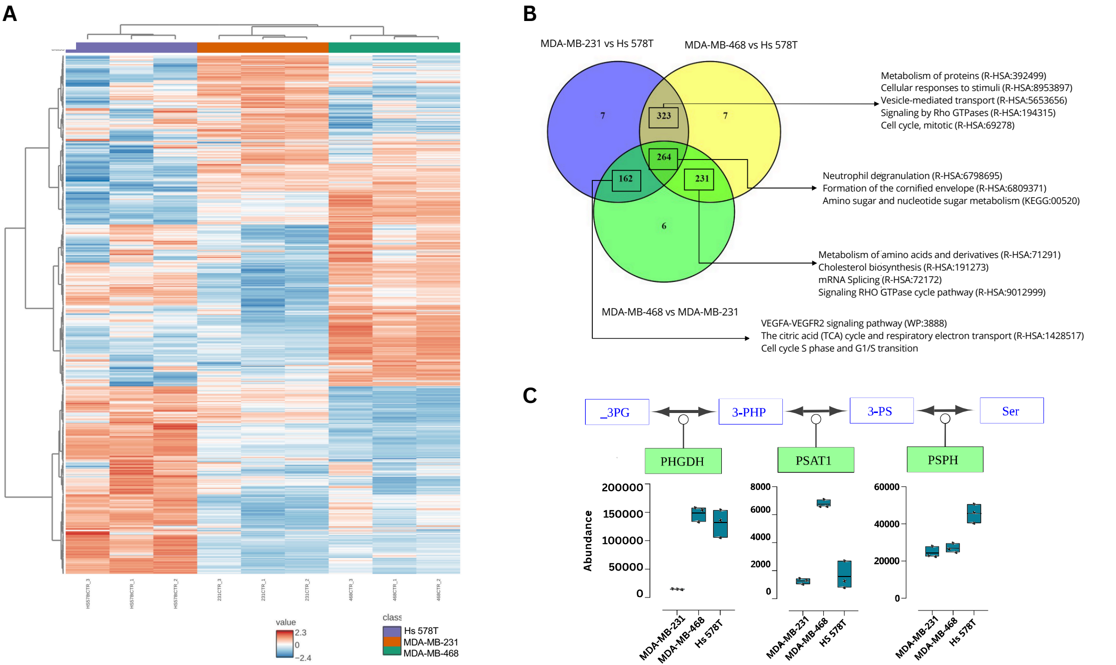

Figure S1. Heterogeneity of untreated TNBC cell line through proteome profiling A. Heatmap of hierarchal clustering analysis performed on protein profiles of TNBC cell panel; Hierarchical clustering was performed on normalized data using Euclidean distance measurement and WARD clustering method option; B. Venn diagram depicting proteins differentiating between cell lines and their involvement in metabolic pathways. Differentially expressed proteins were obtained using ANOVA test (corrected p-value threshold of 0.05) + Tukey's HSD post-hoc test; C. Proteins involved in de novo serine biosynthesis pathway and their expression in TNBC cell panel.

A Overview of intracellular proteome profiling

Intracellular proteome	MDA-MB-231	MDA-MB-468	Hs 578T
Total no. of proteins	2123	1739	1505
Reverse+	84	67	51
FDR dataset %	3.95	3.85	3.39
Final number of proteins	2013	1670	1452

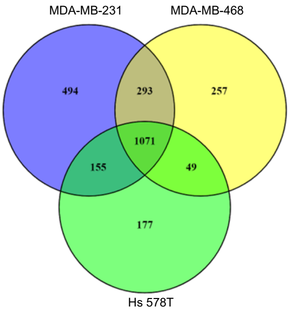

B Functional analysis using GO ontologies

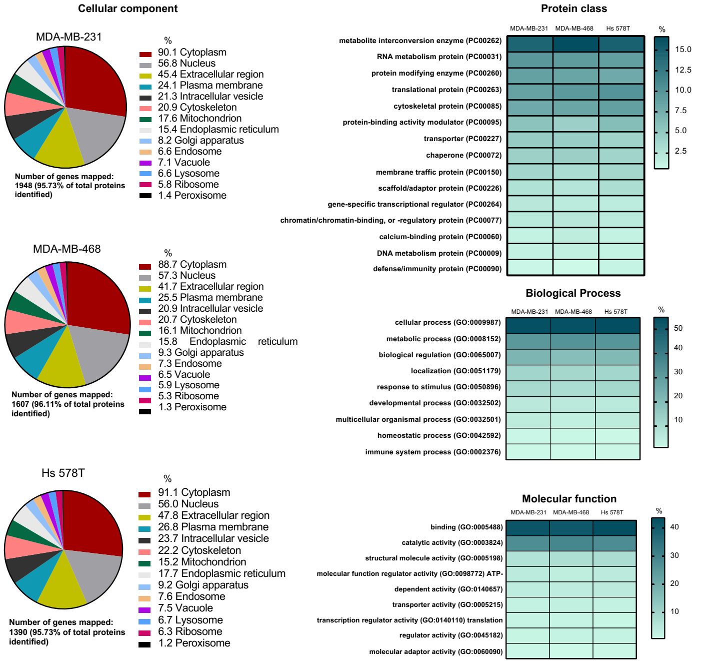

Figure S2. Overview of intracellular proteome profiles: A. Overview of the number of identified proteins and Venn diagram showing common and cell line particular proteins; B. Functional analysis using GO ontologies: cellular component- CC; biological process - BP and molecular function -MF;

A **Overview of secreted proteome profiling**

Secretome	MDA-MB-231	MDA-MB-468	Hs 578T
Total no proteins	112	250	441
Reverse+	3	6	10
FDR dataset %	2.61	2.40	2.32
Final number of proteins	108	243	426

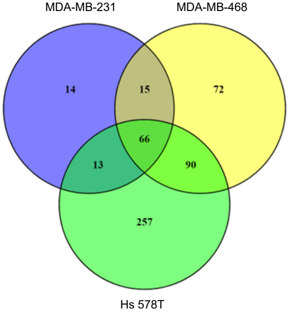

B **Functional analysis using GO ontologies**

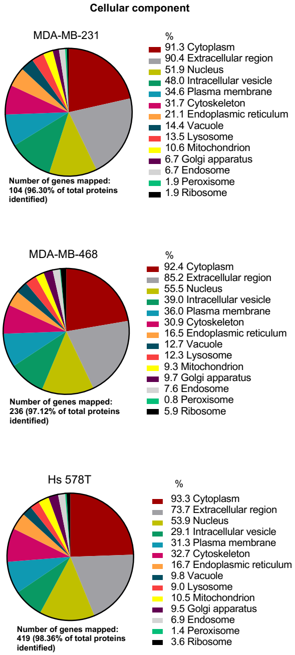

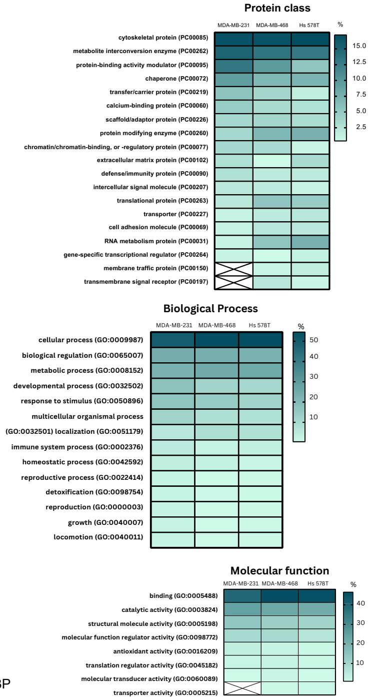

Figure S3. Overview of the secreted proteins profiles: A. Overview of the number of identified proteins and Venn diagram showing common and cell line particular proteins; B. Functional analysis using GO ontologies: cellular component- CC; biological process - BP and molecular function -MF;

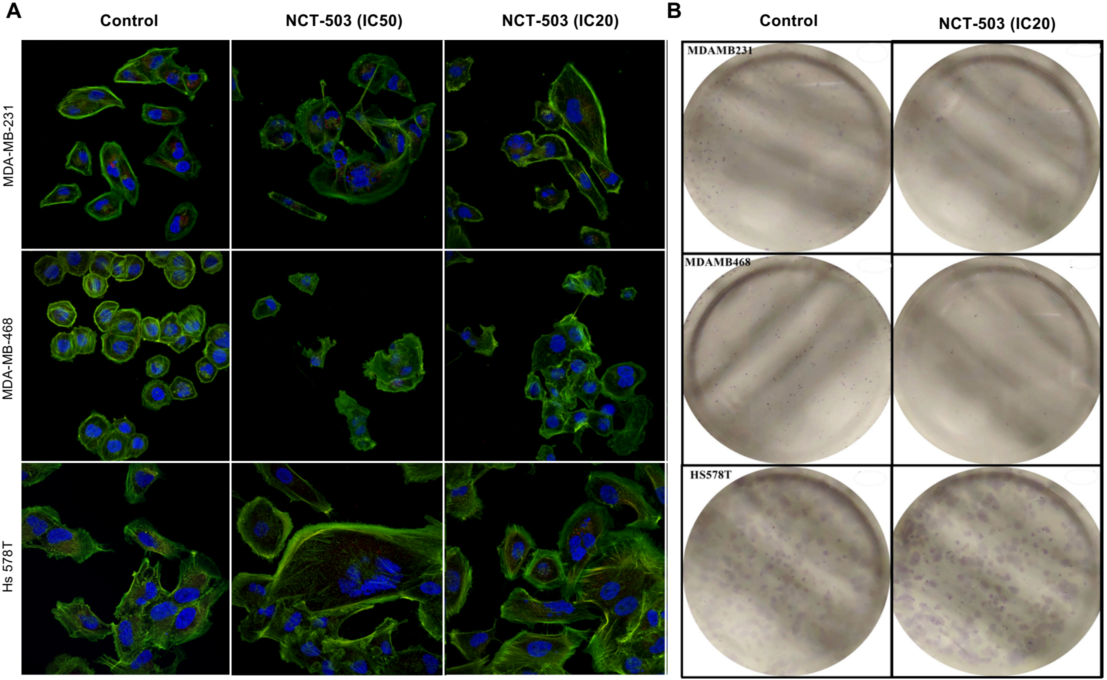

Figure S4. NCT-503 treatment impact on cellular properties of TNBC cell panel A. Impact of NCT-503 treatment on cell morphology at IC50 and IC20 dose, respectively for each TNBC cell line; blue- cell nucleus, green- cytoskeleton morphology; B. Effect of NCT 503 treatment on colony formation abilities of TNBC cell line panel

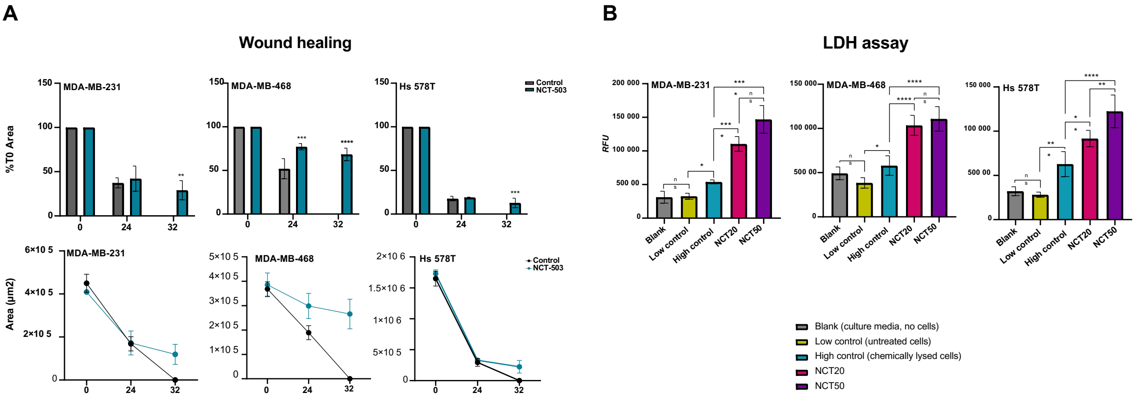

Figure S5 In vitro assays of NCT-503 treatment impact on TNBC cell lines. A. wound healing assay; B. Culture media release LDH assay

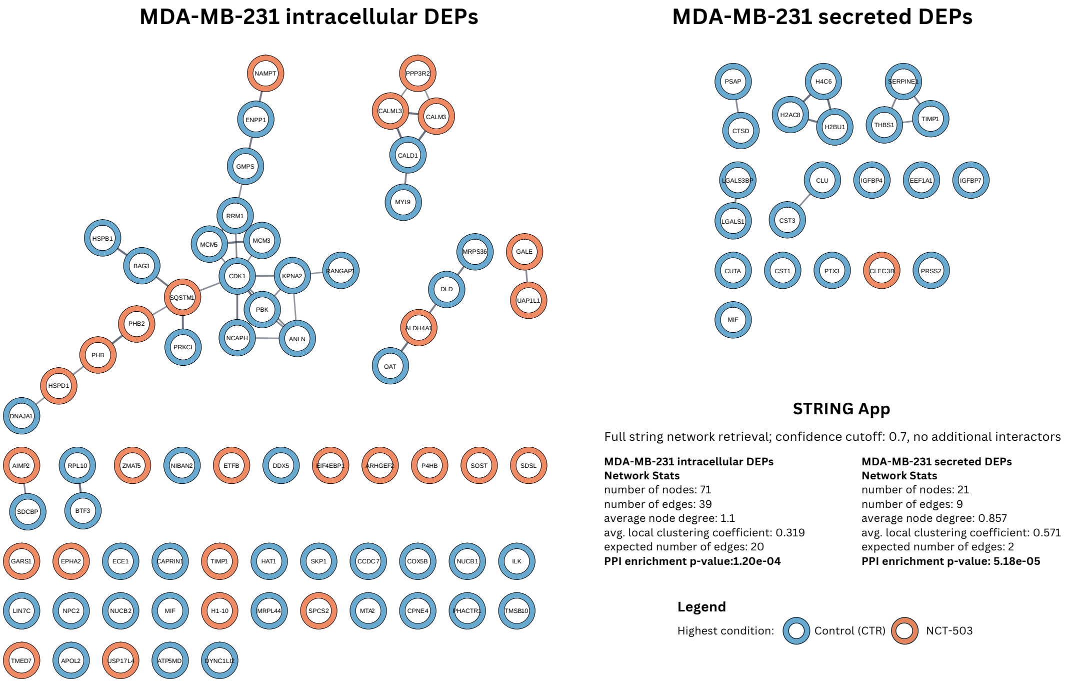

Figure S6. Protein-protein interaction (PPI) network retrieved using StringApp for DEPs induced by NCT-503 treatment in MDA-MB-231 cell line

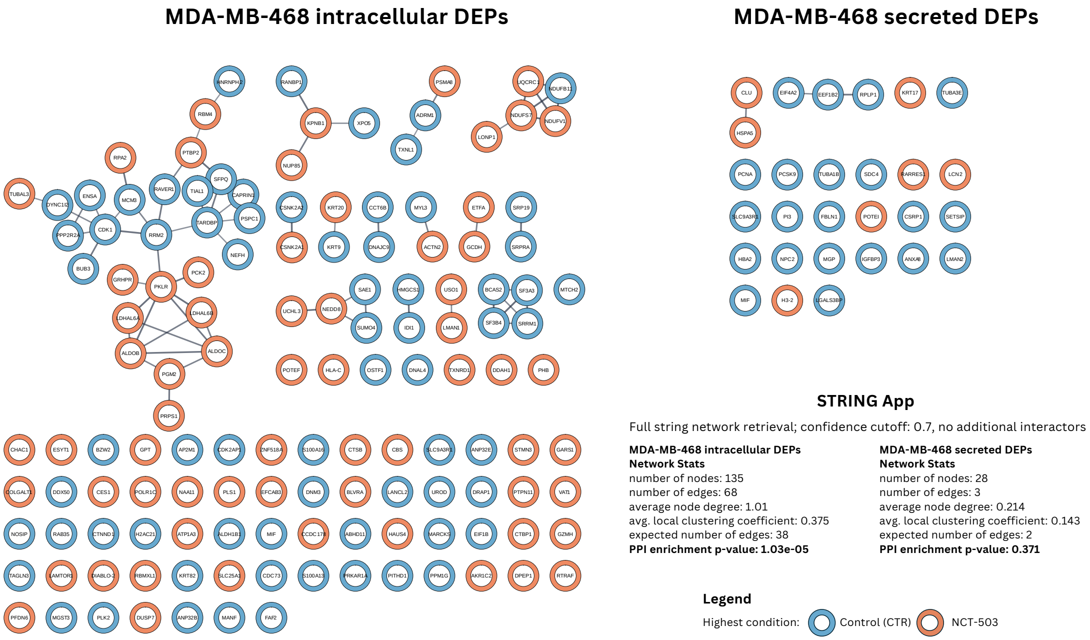

Figure S7. Protein-protein interaction (PPI) network retrieved using StringApp for DEPs induced by NCT-503 treatment in MDA-MB-468 cell line

Hs 578T intracellular DEPs

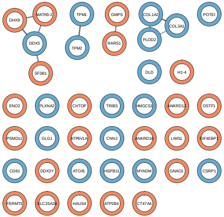

Hs 578T secreted DEPs

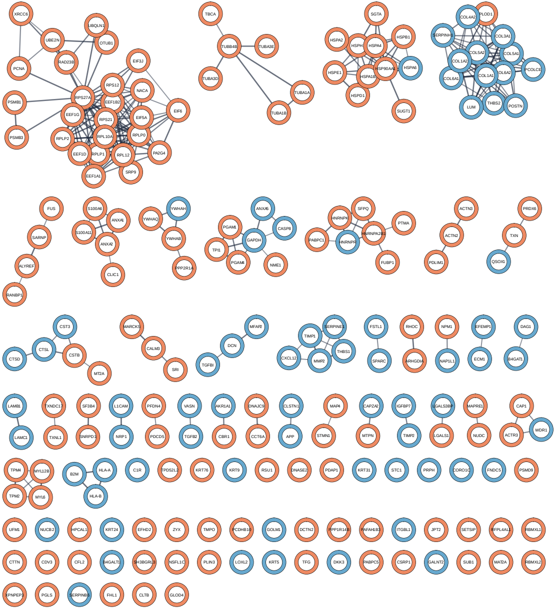

STRING App

Full string network retrieval; confidence cutoff: 0.7, no additional interactors

Hs 578T intracellular DEPs
Network Stats
number of nodes: 40
number of edges: 9
average node degree: 0.45
avg. local clustering coefficient: 0.258
expected number of edges: 3
PPI enrichment p-value: 0.00466

Hs 578T secreted DEPs
Network Stats
number of nodes: 207
number of edges: 481
average node degree: 4.65
avg. local clustering coefficient: 0.465
expected number of edges: 162
PPI enrichment p-value: < 1.0e-16

Legend

Highest condition: Control (CTR) NCT-503

Figure S8. Protein-protein interaction (PPI) network retrieved using StringApp for DEPs induced by NCT-503 treatment in Hs 578T cell line

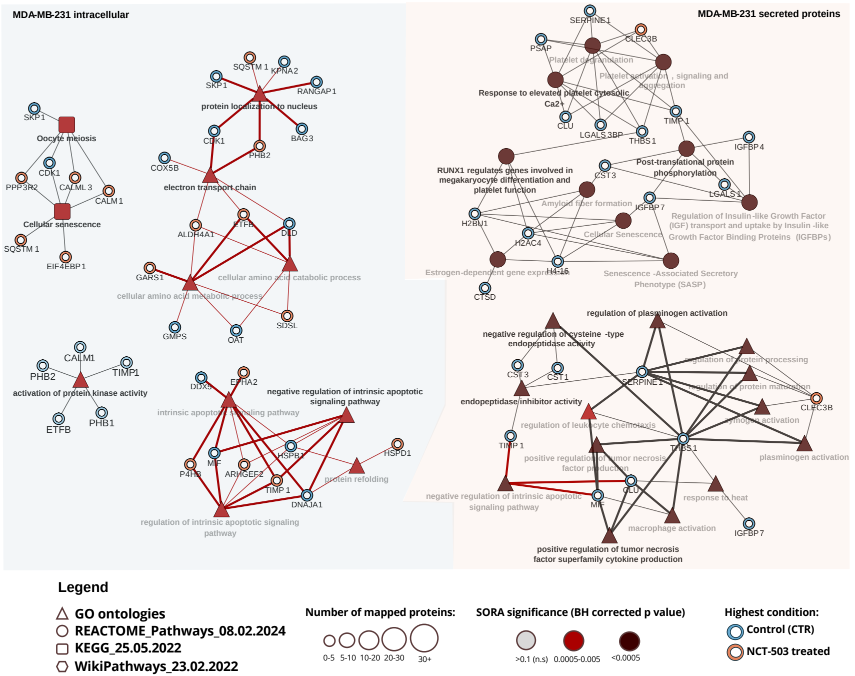

Figure S9. Enrichment analysis of MDA-MB-231 DEPs using ClueGO Enrichment tool

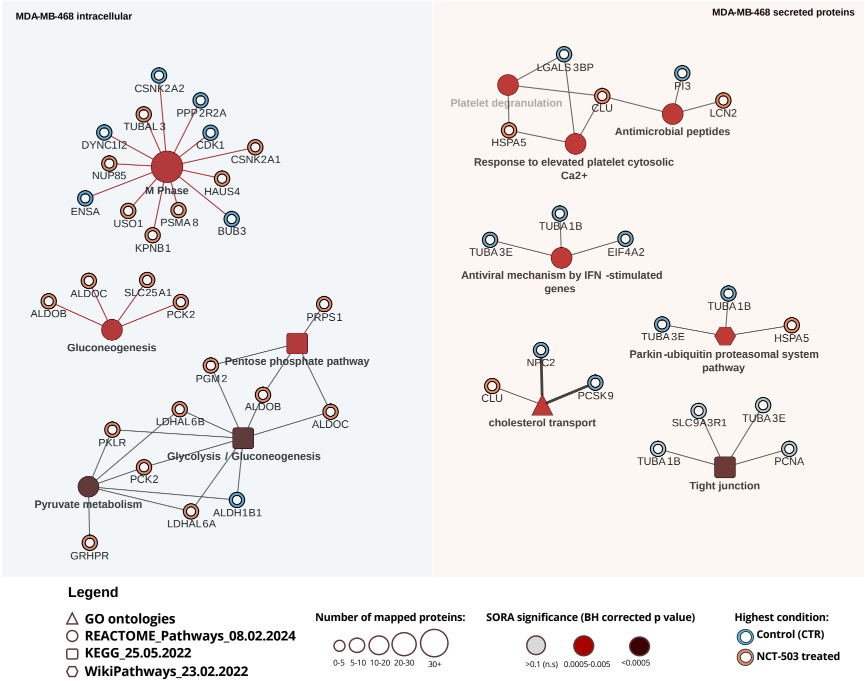

Figure S10. Enrichment analysis of MDA-MB-468 DEPs using ClueGO Enrichment tool

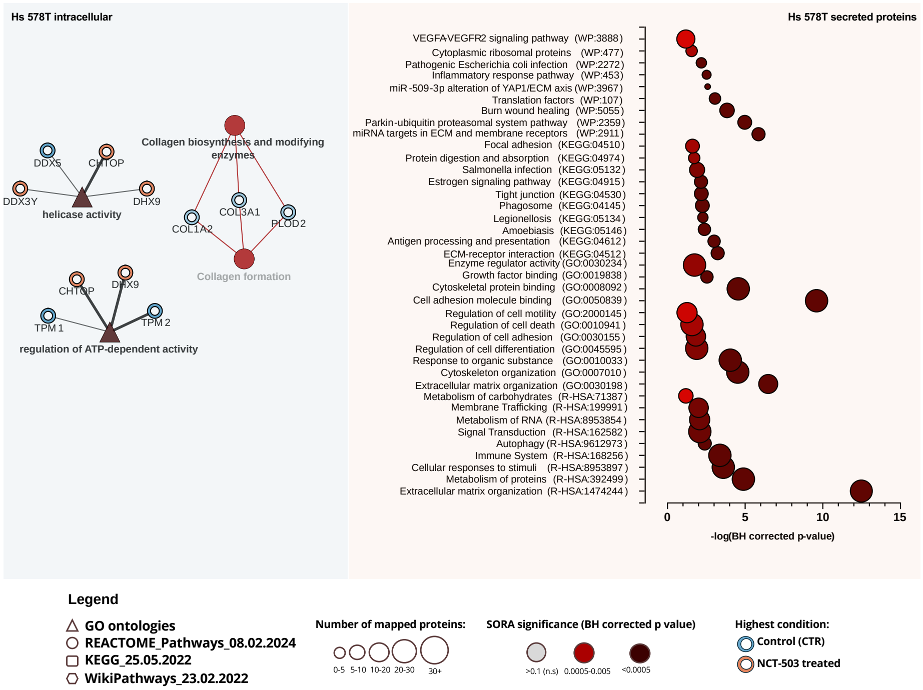

Figure S11. Enrichment analysis of Hs 578T DEPs using ClueGO Enrichment tool

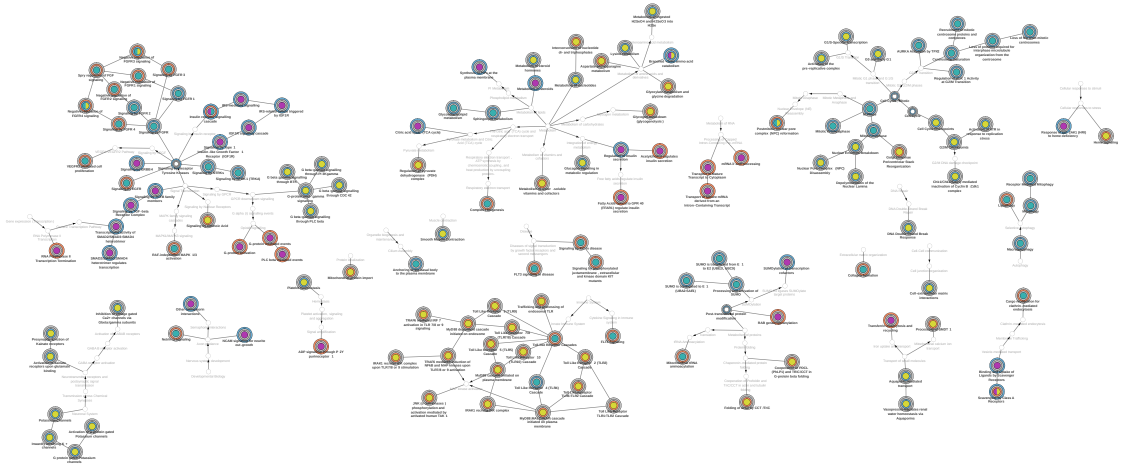

Legend:

Rings (inner to outer):

direction GSEA MDA-MB-231

direction GSEA MDA-MB-468

direction GSEA Hs 578T

Significant Down **Significant Up**

Not significant

MDA-MB-231

MDA-MB-468

Hs 578T

Figure S12. Network representation of Reactome GSEA pathways for the intracellular proteome of TNBC cell lines treated with NCT-503

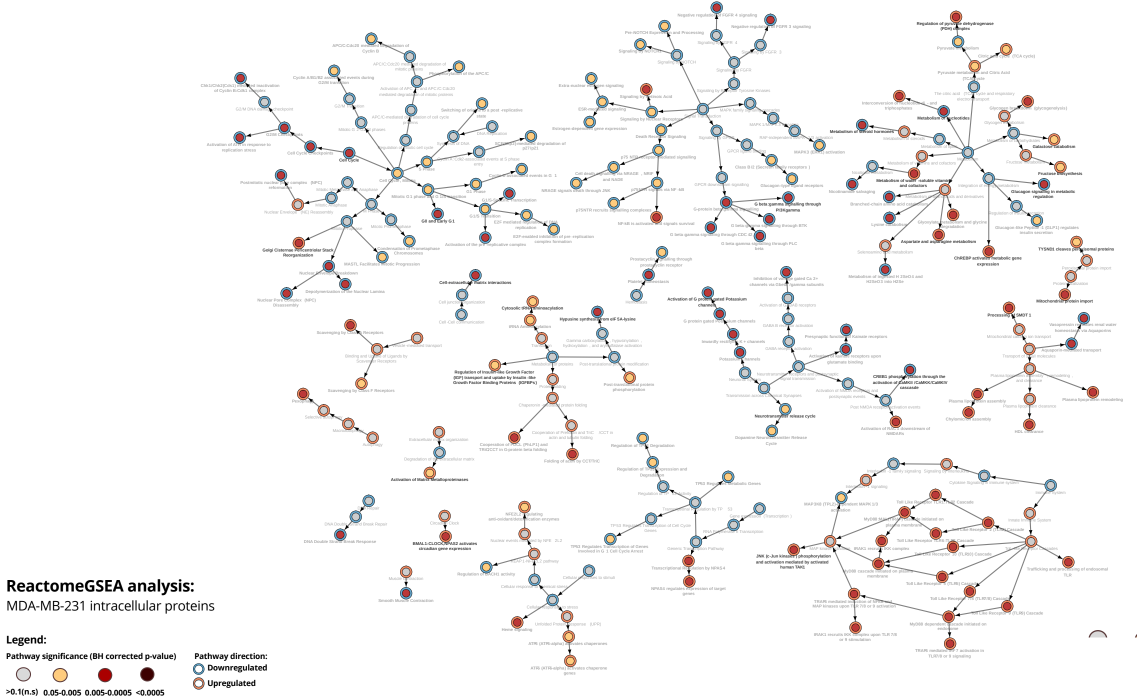

Figure S13. Network representation of ReactomeGSEA analysis performed on the intracellular protein profiles of NCT-503 treatmentand control group of MDA-MB-231

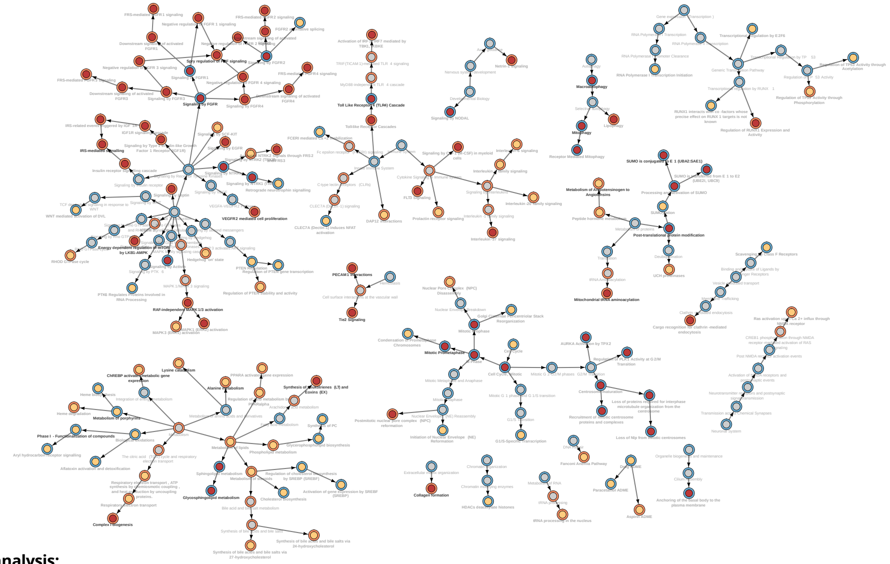

ReactomeGSEA analysis:

MDA-MB-468 intracellular proteins

Legend:

Pathway significance (BH corrected p-value)

>0.1(n.s) 0.05-0.005 0.005-0.0005 <0.0005

Pathway direction:

Downregulated

Upregulated

Figure S14. Network representation of ReactomeGSEA analysis performed on the intracellular protein profiles of NCT-503 treatmentand control group of MDA-MB-468

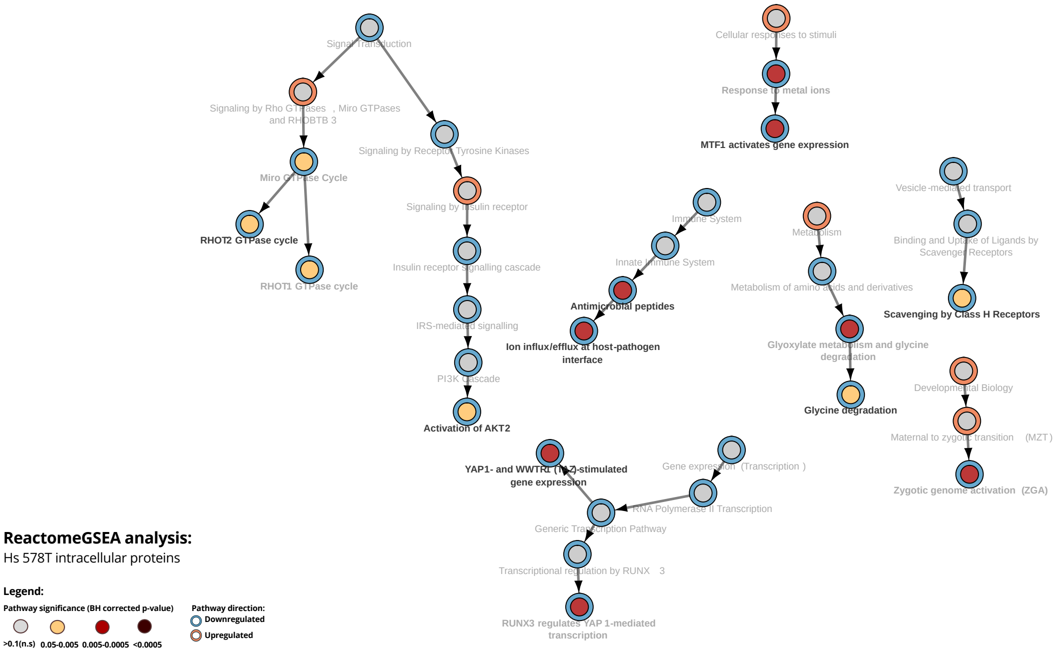

Figure S15. Network representation of ReactomeGSEA analysis performed on the intracellular protein profiles of NCT-503 treatmentand control group of Hs 578T

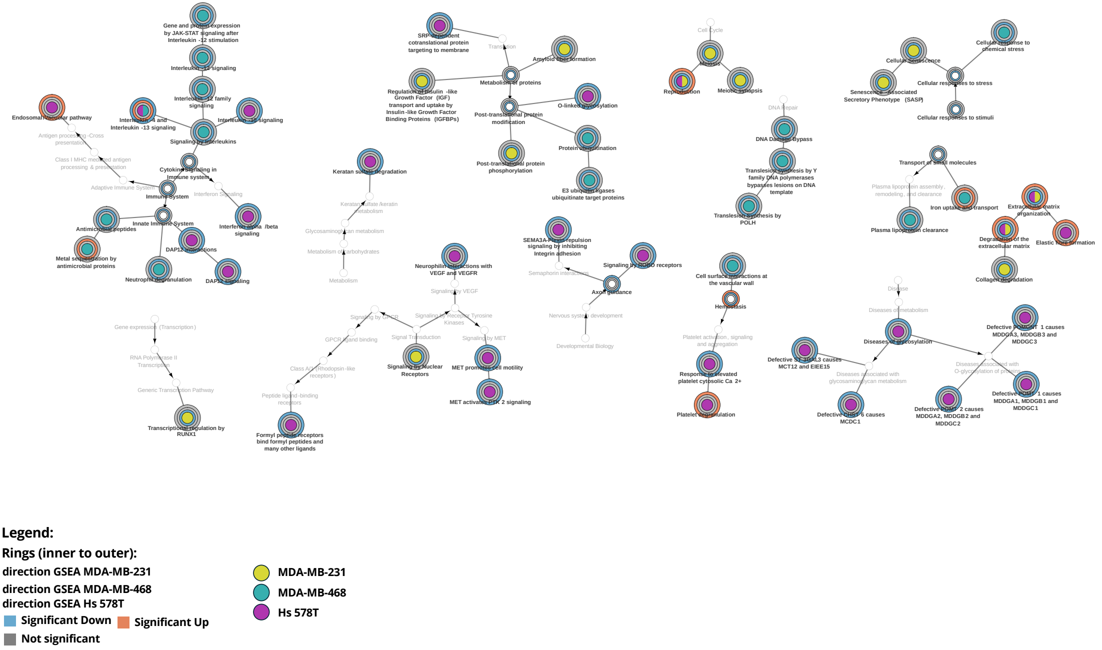

Figure S16. Network representation of Reactome GSEA pathways for the secreted proteome of TNBC cell lines treated with NCT-503

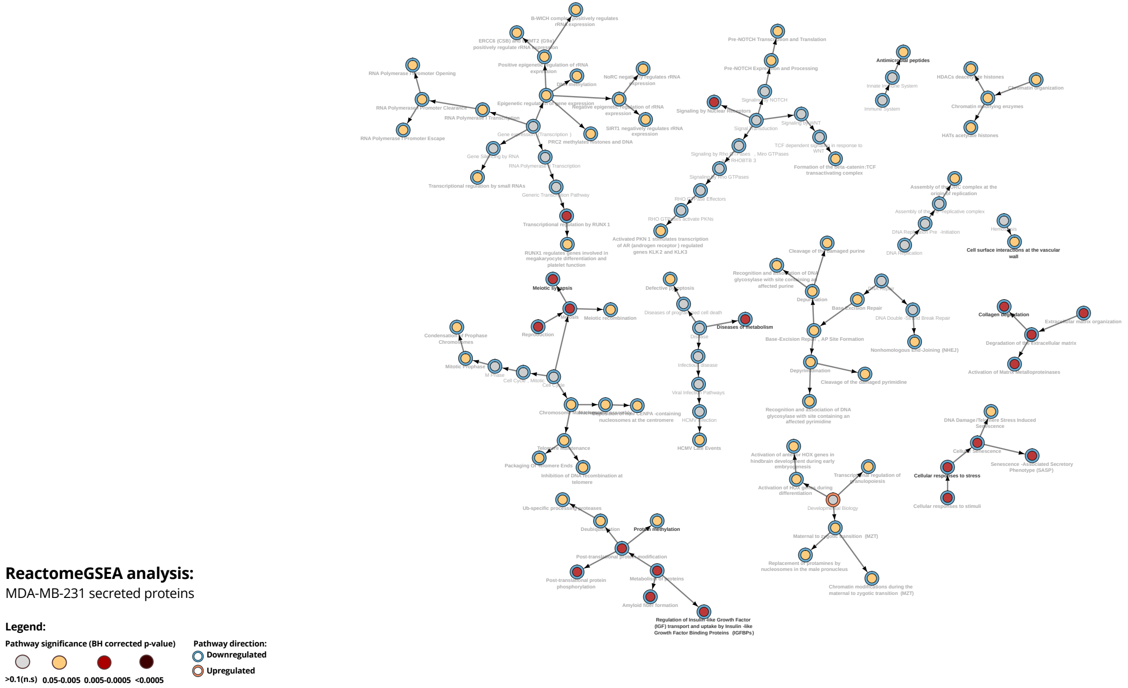

Figure S17. Network representation of ReactomeGSEA analysis performed on the secreted protein profiles of NCT-503 treatment and control group of MDA-MB-231

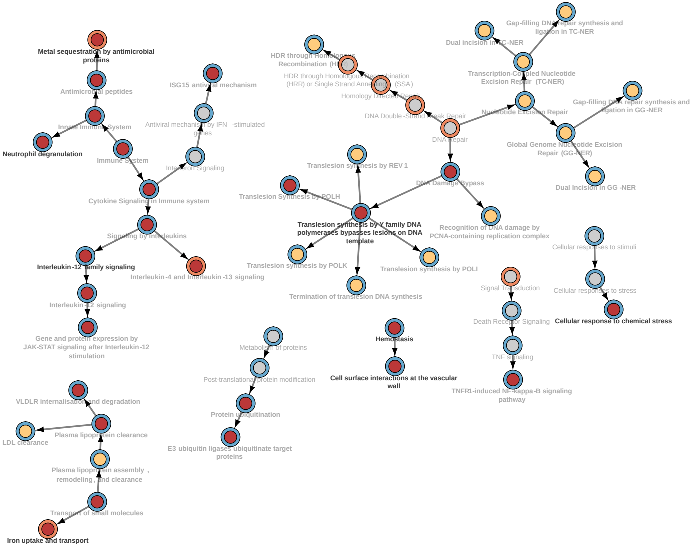

ReactomeGSEA analysis:

MDA-MB-468 secreted proteins

Legend:

Pathway significance (BH corrected p-value): >0.1(n.s) | 0.05-0.005 | 0.005-0.0005 | <0.0005

Pathway direction: Downregulated | Upregulated

Figure S18. Network representation of ReactomeGSEA analysis performed on the secreted protein profiles of NCT-503 treatment and control group of MDA-MB-468

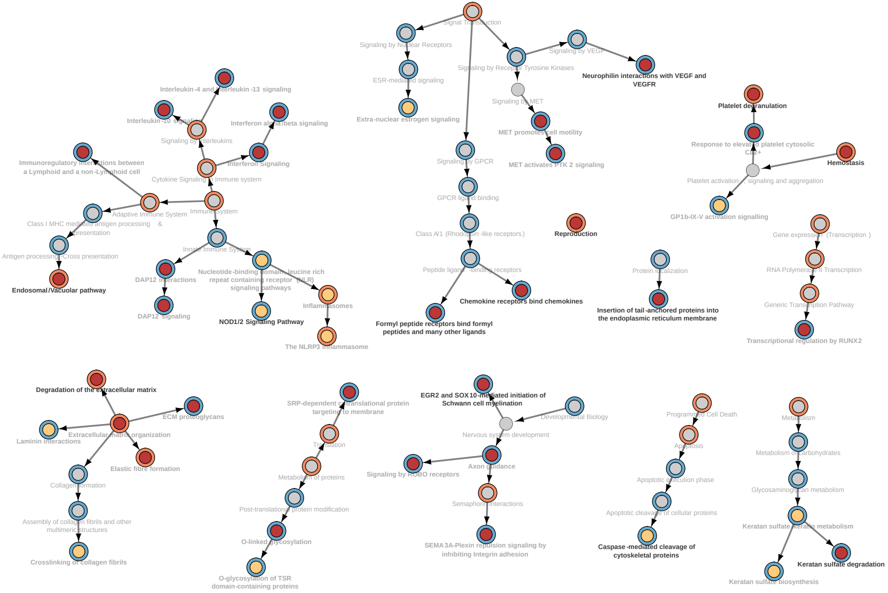

ReactomeGSEA analysis:

Hs 578T secreted proteins

Legend:

Pathway significance (BH corrected p-value)

>0.1(n.s) 0.05-0.005 0.005-0.0005 <0.0005

Pathway direction:

Downregulated

Upregulated

Figure S19. Network representation of ReactomeGSEA analysis performed on the secreted protein profiles of NCT-503 treatment and control group of Hs 578T
