# Supplementary material for: Cellular Responses Induced by NCT-503 Treatment on Triple-Negative Breast Cancer Cell Lines: A Proteomics Approach
Source: Biomedicines. 2024 May 14;12(5):1087. doi: 10.3390/biomedicines12051087 (PMC11117597; doi:10.3390/biomedicines12051087)
Supplement: Supplementary file 1 [file biomedicines-12-01087-s001.zip › Supplementary Information S1-S2.pdf]

biomedicines

MDPI

Supplementary Information

Cellular Responses Induced by NCT-503 Treatment on Triple-Negative Breast Cancer Cell Lines: A Proteomics Approach

Ioana-Ecaterina Pralea [1,2], Radu-Cristian Moldovan [1], Adrian-Bogdan Țigu [3], Cristian-Silviu Moldovan [4], Eva Fischer-Fodor [5] and Cristina-Adela Iuga [1,2,*]

[1] Department of Proteomics and Metabolomics, Research Center for Advanced Medicine–MedFuture, "Iuliu Hațieganu" University of Medicine and Pharmacy Cluj-Napoca, Louis Pasteur Street 4-6, 400349 Cluj-Napoca, Romania; pralea.ioana@umfcluj.ro (I.-E.P.) moldovan.radu@umfcluj.ro (R.-C.M.)

[2] Department of Pharmaceutical Analysis, Faculty of Pharmacy, "Iuliu Hațieganu" University of Medicine and Pharmacy, Louis Pasteur Street 6, 400349 Cluj-Napoca, Romania

[3] Department of Translational Medicine, Research Center for Advanced Medicine–MedFuture, "Iuliu Hațieganu" University of Medicine and Pharmacy Cluj-Napoca, Louis Pasteur Street 6, 400349 Cluj-Napoca, Romania; bogdan.tigu@umfcluj.ro

[4] Department of BioNanoPhysics, Research Center for Advanced Medicine–MedFuture, "Iuliu Hațieganu" University of Medicine and Pharmacy Cluj-Napoca, Louis Pasteur Street 6, 400349 Cluj-Napoca, Romania; moldovan.cristian1994@gmail.com

[5] Tumor Biology Department, Institute of Oncology "Prof. Dr. Ion Chiricuță", 400015 Cluj-Napoca, Romania; fischer.eva@iocn.ro

* Correspondence: iugac@umfcluj.ro

Contents

Supplementary information S1. Overview of the intracellular and secreted proteome profiles analysis 1

Supplementary information S2. TNBC cell line response to NCT-503 treatment 2

S2.1 MDA-MB-231 response to NCT-503 treatment 2

S2.2 MDA-MB-468 response to NCT-503 treatment 4

S2.3 Hs 578T response to NCT-503 treatment 5

Supplementary information S1. Overview of the intracellular and secreted proteome profiles analysis

Intracellular proteome profiling data consisted of 2123 proteins in the case of MDA-MB-231, 1739 and 1505 in the case of MDA-MB-468 and Hs 578T, respectively, with quantitative values based on 47042, 44111 and 35800 identified peptides. The calculated FDR for each dataset was below 4%. 1072 proteins were common to all TNBC cell lines, also, there were cell line specific proteins (Figure S2A). The profiling of secreted proteins consisted of a total of 112 proteins in the case of MDA-MB-231 while 250 and 441 proteins were found in the culture media of MDA-MB-468 and Hs 578T, respectively (Figure S3A), 66 of which were shared between the three cell lines.

Proteins were mapped according to their location using SubcellulaRVis online tool (Figures S2B, S3B). The protocol implemented for intracellular proteome analysis achieved similar performance regardless of the cell line: most of the proteins were mapped to the cytoplasm (90%) and nucleus (57%); plasma membrane, intracellular vesicle and the cytoskeleton were also well represented (>20%) (Figure S2B); on the other end, the

proteins mapped to the lysosome, ribosome and peroxisome represented by less than 6% of the proteome profile. In the case of the protocol used for secreted proteins analysis, functional analysis mapped most of the identified proteins to the cytoplasm and extracellular region (92% and 83% respectively) with no significant differences between the cell lines. Nucleus was represented by 54% of the proteins, while 34% of proteins were attributed to plasma and 32% to the cytoskeleton. Differences between the TNBC cell lines regarding the percent of proteins mapped to a certain cell com-ponent were observed for the endoplasmic reticulum (21% for MDA-MB-231 and 17% for the other two cell lines), lysosome (13% for the MDAs and 9% for Hs 578T), Golgi apparatus (7% for MDA-MB-231 and 10% in the case of MDA-MB-468 and Hs 578T) and for the ribosome (2% MDA-MB-231, 4% Hs 578T and 6% MDA-MB-468).

Panther Functional classification tool was further used to group proteins identified in each cell line according to the biological processes (BP), molecular function (MF) and protein class (PC) ontologies (Figure S2B and S3B). No significant differences between the percent of genes classified to a category over the total number of genes were observed between the cell lines. The intracellular proteome profile was mostly represented by biological processes involved in the cellular (GO:0009987) and metabolic (GO:0008152) processes (around 54% and 32%, respectively). Proteins implicated in biological regulation (GO:0065007), localization (GO:0051179) and response to stimulus (GO:0050896) were presented in the dataset (21%, 12% and 11%, respectively). The molecular functions of the intracellular proteins were mainly reported as binding (GO:0005488) and catalytic activity (GO:0003824) (42% and 28%). In terms of PC, functional analysis results emphasized that the intracellular proteome profile was rich in proteins implicated in metabolite interconversion (PC00262) - 15%, RNA metabolism (PC00031) - 9%, translation (PC00263) - 9% and cytoskeletal proteins - 8%. Functional analysis results of the secreted proteins are shown in Figure S3B. Cellular process (GO:0009987), biological regulation (GO:0065007) and metabolic process (GO:0008152) represented top terms describing more than 20% of the secretome profile obtained. Molecular function ontology highlighted binding (GO:0005488) and catalytic activity (GO:0003824) terms (>20%) while PC ontology described the secreted proteins as mainly cytoskeletal (PC00085) -17%, implicated in metabolite interconversion (PC00262) – 14% and with protein-binding activity modulator (PC00095).

Supplementary information S2. TNBC cell line response to NCT-503 treatment

S2.1 MDA-MB-231 response to NCT-503 treatment

After 48h exposure to NCT-503, the cytotoxic potential on MDA-MB-231 was evaluated by MTT Assay (Figure 2A). The IC50 value determined for MDA-MB-231 was 93.4 ±14.0 μM expressed as mean and standard error. Both the IC20 and IC50 doses were used to evaluate the morphological changes in MDA-MB-231 cell line. NCT-503 treatment at IC20 (34.8±8.7 μM) induced multilobulate nuclei and changes in the morphology associated with a tendency to shift into round shaped cells, indicative of acute cellular stress; while at IC50, NCT-503 treatment caused fragmented nuclei in multiple cells (Figure S4A). Also, the colony formation process in MDA-MB-231 was inhibited by NCT-503 treatment after 14 days of incubation (Figure S4B). Almost 80% of the original wound was closed after compared to the control group after 32h indicating that NCT-503 impaired the wound healing capability of MDA-MB-231. Significant LDH enzyme release was observed in the NCT-503 treated MDA-MB-231 cell line also, a dose-dependent enzyme release was confirmed (Figure S5).

NCT-503 treatment significantly altered the abundance of 71 proteins (regarded as differently expressed proteins: DEPs) attributed to the intracellular proteome of MDA-MB-231 cell line (Table S2), mostly being up-regulated (45 proteins). On the other hand, on the secreted proteins of the same cell line, treatment affected the abundance of 21 proteins (Figure 2C, Table S3). StringApp tool was used to retrieve PPI networks of the significant different abundant proteins after NCT-503 treatment (Figure S6). In the case of

the MDA-MB-231 cell line, the PPI networks indicated that these proteins have more interactions among themselves being at least partially biologically connected as a group (intracellular PPI enrichment p-value: 1.20e-04 and secretome PPI enrichment p-value: 5.18e-05).

Enrichment analysis was conducted using multiple tools in order to address the limitations of enrichment analysis (namely, how enrichment is conducted in a particular software, version of the database available with the enrichment tool, the curated status of the enrichment databeses): ClueGO and Cluepedia (depicted in the Figure S9), StringApp enrichment, Gprofiler and EnrichR (Tables S4, S5). These enrichment analysis tools were used with several common ontologies such as GO ontologies and ReactomeDB, KEGG and Wikipathways pathways databases., also, particular to a single tool (EnrichR), was the MsigDB Hallmarks database (Table S6).

ClueGO enrichment analysis associated MDA-MB-231 intracellular DEPs (Figure S9) to several GO biological processes namely, electron transport chain (GO:0022900; BH corrected p-value: 1.80e-03; 6 proteins), protein localization to nucleus (GO:0034504; BH corrected p value: 2.51e-03; 7 proteins) and negative regulation of intrinsic apoptotic signaling pathway (GO:2001243; BH corrected p value: 1.88e-03; 5 proteins). DEPs attributed to GO MF activation of protein kinase activity (GO:0032147, BH corrected p-value: 2.96e-03; 5 proteins) were found downregulated in NCT-503 treated vs. the CTR group. Using KEGG database, intracellular DEPs were classified to cellular senescence term (KEGG:04218, BH corrected p-value: 2.28e-03; 6 proteins) and oocyte meiosis (KEGG:04114, BH corrected p-value: 4.50e-03; 5 proteins) with most of proteins showing highest abundance in NCT-503 treated group.

Most of the NCT-503 induced DEPs in the secretome of MDA-MB-231 were downregulated and top terms attributed by ClueGO analysis involved hemostasis (R-HSA:76005 response to elevated platelet cytosolic Ca2+, BH corrected p-value: 6.64e-09, 7 proteins), post-translational protein modifications (R-HSA:8957275 post-translational protein phosphorylation, BH corrected p-value: 1.91e-06, 5 proteins) and transcriptional regulation by RUNX1 (R-HSA:8936459 RUNX1 regulates genes involved in megakaryocyte differentiation and platelet function, BH corrected p-value: 2.59e-05, 4 proteins). BP GO ontology results showed enrich terms implicated in protein processing (GO:0010755 regulation of plasminogen activation, BH corrected p-value: 1.20e-05; 3 proteins) and regulation of cytokine production (GO:1903557 positive regulation of tumor necrosis factor superfamily cytokine production, BH corrected p-value: 3.94e-04, 3 proteins) and endopeptidase inhibitor activity (GO:0004866, BH corrected p-value: 2.44e-04, 3 proteins).

GSEA analysis performed on the MDA-MB-231 cell line (Figure S13, Tables S7) highlighted several pathway modifications as response to the NCT-503 treatment: i. Cell cycle pathway (R-HSA:1640170, 185 proteins) and associated pathways cell cycle checkpoints (R-HSA:69620, 97 proteins), G2/M checkpoints (R-HSA:69481, 70 proteins) and G0 and early G1 phase pathway (R-HSA:1538133, 6 proteins) were found all downregulated based on the intracellular proteome; ii. Metabolism of nucleotides (R-HSA:15869, 39 proteins) and steroid hormones (R-HSA:1960713, 3 proteins) were also found to be downregulated as response to NCT-503 treatment; iii. The catabolism of lysine (R-HSA:71064, 4 proteins) and branched-chain amino acids (R-HSA:70895, 7 proteins) were also significantly downregulated after treatment as opposed to aspartate and asparagine metabolism (R-HSA:8963693, 4 proteins) and glyoxylate metabolism and glycine degradation (R-HSA:389661, 9 proteins) found up-regulated; iv. Cell-extracellular matrix interactions pathway was also found downregulated as response to NCT-503 (R-HSA:446353, 10 proteins); v. several signal transduction pathways were also found influenced by treatment: down regulation of signaling by FGFR 3 and 4 pathways (R-HSA:5654733 and R-HSA:5654732, 7 proteins), up-regulation of Signaling by retinoic acid (R-HSA:5362517, 7 proteins) and NF-kB is activated and signals survival (R-HSA:5362517, 7 proteins) pathways.

GSEA analysis performed on the secreted proteins after NCT-503 treatment (Figure S17, Tables S8) showed down-regulation of pathways implicated in extracellular matrix organization (R-HSA:1474244, 8 proteins) and cellular response to stimuli (R-HSA:8953897, 21 proteins).

S2.2 MDA-MB-468 response to NCT-503 treatment

In the MDA-MB-468 cell line, NCT-503 had an IC50 of 20.2±2.8 μM expressed as mean and standard error of 3 independent MTT assay experiments (Figure 2A). The morphological evaluation highlighted the tendency of a less defined cytoplasmic membrane and irregular shape of MDA-MB-468 cell lines when treated with NCT503 at IC20 (5.7±1.6 μM). Additionally, the IC_{50} dose significantly affected overall viability characterized by fewer cells in the microscopical field and fragmented cytoskeleton with less defined actin fibers (Figure 2B, Figure S4A). The colony formation and cell migration were significantly affected by treatment with less than 50% of the original wound being closed after 32h compared to the untreated group (Figure S4B). MDA-MB-468 displayed a significant LDH release in the media although a dose-dependent effect could not be confirmed (Supplementary Figure S5).

A total of 138 intracellular DEPs were induced by NCT-503 treatment in the case of MDA-MB-468 cell line (Figure 2C, Table S2). PPI network retrieved for MDA-MB-468 intracellular DEPs showed interactions among themselves while the DEPs obtained from the cell culture media secreted proteins profile seem to be a random set of proteins not very well connected (Figure S7).

ClueGO analysis (Figure S10, Table S4) using Reactome database highlighted glucose metabolism pathway (R-HSA:70263 Gluconeogenesis, BH corrected p-value: 4.94e-03, 4 proteins) and cell cycle pathway (R-HSA:68886 M Phase, BH corrected p-value: 4.67e-03, 13 proteins) terms enriched through the MDA-MB-468 intracellular DEPs. Results were confirmed using KEGG database where pathways closely related were attributed to intracellular DEPs: Glycolysis / Gluconeogenesis (KEGG:00010, BH corrected p-value: 7.02e-06, 8 proteins), Pentose phosphate pathway (KEGG:00030, BH corrected p-value: 1.86e-04, 4 proteins), Pyruvate metabolism (KEGG:00620, BH corrected p-value: 5.41e-06, 6 proteins). 29 DEPs were found in the secretome profile of NCT-503 treatment vs control cases of MDA-MB-468 cell line: most of them with lower abundant. SORA analysis using Reactome Database revealed secreted DEPs involved in immune response (R-HSA:1169410 Antiviral mechanism by IFN-stimulated genes, BH corrected p-value: 3.07e-03, 3 proteins and R-HSA:6803157 Antimicrobial peptides, BH corrected p-value: 3.37e-03, 3 proteins) and Platelet degranulation (R-HSA:76005 Response to elevated platelet cytosolic Ca2+, BH corrected p-value: 3.03e-03, 3 proteins). GO ontologies emphasized cholesterol transport (GO:0030301, BH corrected p-value: 6.94e-04, 3 proteins) and low-density lipoprotein particle receptor binding (GO:0050750, BH corrected p-value: 1.00e-05, 3 proteins) terms for the secreted DEPs; while KEGG and Wikipathways associated pathways were Tight junction pathway (KEGG:04530, BH corrected p-value: 3.46e-04, 4 proteins) and Parkin-ubiquitin proteasomal system pathway (WP:2359, BH corrected p-value: 6.09e-04, 3 proteins).

GSEA analysis performed on MDA-MB-468 intracellular proteome profile (Figure S14, Table S9) showed: i. downregulation of cell cycle, mitotic (R-HSA:69278, 152 proteins) and M phase associated pathway (R-HSA:68886, 126 proteins); ii. as response to treatment, the MDA-MB-468 cell line significantly downregulated its sphingolipid metabolism (R-HSA:428157, 7 proteins) and up-regulates the collagen synthesis (R-HSA:1474290, 6 proteins); iii. macro autophagy associated pathways were found to be down-regulated after treatment (R-HSA:1632852, 43 proteins); iii. signaling by tyrosine-kinases pathways were affected in MDA-MB-468 after treatment: augmentation of VEGFR2 mediated cell proliferation (R-HSA:5218921, 3 proteins) signaling by EGFR (R-HSA:177929, 5 proteins) and downregulation of signaling by FGFR (R-HSA:190236, 17 proteins) and NTRK (R-HSA:166520, 26 proteins); iv. Signaling pathway RAF-independent MAPK1/3activation was found up-regulated (R-HSA:112409, 5 proteins); based on the secreted protein profile

(Figure S18, Table S10), GSEA analysis highlighted the downregulation of immune system associated pathways (R-HSA:168256, 80 proteins), transport of small molecules (R-HSA:382551, 15 proteins) and cell surface interactions at the vascular wall (R-HSA:202733, 6 proteins). Up-regulation of iron uptake and transport (R-HSA:917937, 3 proteins) was seen as response to the NCT-503 treatment.

S2.3 Hs 578T response to NCT-503 treatment

NCT-503 treatment affected cell proliferation in Hs 578T achieving an IC_{50} of 76.6±3.2 μM (Figure 2A). Morphological changes in Hs 578T cell line when treated with NCT-503 at IC_{20} (19.2±7.9 μM) consisted of visible gaps between the cells, fragmented nuclei indicating a cell death initiation (Figure 2B, Figure S4A). Furthermore, these cells showed significant morphological changes through gigantic catastrophe with enlarged cells in the colony (Figure S4B). Hs 578T colony formation was not influenced by the NCT-503 treatment, both control and treated group generated colonies after 14 days. Moreover, the migration was also not inhibited, more than 80% of the wound was closed after 32h. The NCT-503 treated cells presented high LDH release in the media, the effect being dose-dependent manner, being highly significant compared to the control (Figure S5).

NCT-503 treatment of the Hs 578T cell line seems to have little impact on the intracellular proteome: although well connected as a functional group (PPI network analysis p-value: 0.00466) (Figure S8), only 40 DEPs were found using univariate analysis (Figure 2C, Table S2). The opposite was observed in the case of the secreted proteins where 208 DEPs (Figure 2C, Table S3), biologically connected (PPI network analysis p-value < $1.0e^{-16}$), were identified.

SORA analysis (Figure S11) using ClueGO highlighted that top associated terms with the intracellular DEPs (Table S4) were related to collagen biosynthesis (R-HSA:1650814, BH corrected p-value: $2.80e^{-03}$, 3 proteins). Hs 578T secreted DEPs enriched terms analyzed using Reactome database emphasized pathways corresponding to extracellular matrix organization (R-HSA:1474244, BH corrected p-value: $1.97e^{-13}$, 29 proteins), metabolism of proteins (R-HSA:392499, BH corrected p-value: $1.69e^{-06}$, 60 proteins), cellular responses to stimuli (R-HSA:8953897, BH corrected p-value: $2.57e^{-05}$, 30 proteins), immune system response (R-HSA:168256, BH corrected p-value: $4.09e^{-05}$, 55 proteins), autophagy (R-HSA:9612973, BH corrected p-value: $3.17e^{-04}$, 10 proteins), signal transduction (R-HSA:162582, BH corrected p-value: $6.12e^{-04}$, 60 proteins) and membrane trafficking (R-HSA:199991, BH corrected p-value: $7.32e^{-04}$, 22 proteins). Top enriched terms described using GO BP ontology were extracellular matrix organization (GO:0030198, BH corrected p-value: $5.87e^{-08}$, 21 proteins), cytoskeleton organization (GO:0007010, BH corrected p-value: $3.60e^{-06}$, 43 proteins), response to organic substance (GO:0010033, BH corrected p-value: $9.93e^{-06}$, 66 proteins) and regulation of cell differentiation (GO:0045595, BH corrected p-value: $9.27e^{-04}$, 36 proteins), cell adhesion (GO:0030155, BH corrected p-value: $1.06e^{-03}$, 22 proteins), cell death (GO:0010941, BH corrected p-value: $1.78e^{-03}$, 36 proteins) and cell motility (GO:2000145, BH corrected p-value: $3.48e^{-03}$, 24 proteins). The general image of the secreted DEPs metabolic implications was drew by GO MF ontology with top terms involved in cell adhesion molecule binding (GO:0050839, BH corrected p-value: $8.49e^{-11}$, 31 proteins), cytoskeletal protein binding (GO:0008092, BH corrected p-value: $3.41e^{-06}$, 32 proteins) and growth factor binding (GO:0019838, BH corrected p-value: $2.35e^{-04}$, 9 proteins). ECM-receptor interaction (KEGG:04512, BH corrected p-value: $5.48e^{-05}$, 10 proteins) and antigen processing and presentation (KEGG:04612, BH corrected p-value: $8.87e^{-05}$, 9 proteins) were enriched terms ascribed to the Hs 578T secretome DEPs using KEGG database. Lastly, WikiPathways added miRNA targets in ECM and membrane receptors (WP:2911, BH corrected p-value: $2.19e^{-07}$, 10 proteins) and Parkin-ubiquitin proteasomal system pathway (WP:2359, BH corrected p-value: $1.41e^{-06}$, 11 proteins) terms to the ORA of secreted Hs 578T DEPs (Table S4).

In the case of Hs 578T, GSEA analysis performed on the intracellular proteome profiling (Figure S15, Table S11) showed that after NCT-503 treatment, the cell significantly downregulates its metabolism of steroids (R-HSA:8957322, 12 proteins) and synthesis of phosphatidylinositol 4-phosphates at the plasma membrane (R-HSA:1660499, 3 proteins), branched-chain amino acid catabolism (R-HSA:70895, 6 proteins), regulation of insulin secretion (R-HSA:422356, 15 proteins) and the citric acid cycle (TCA cycle) pathway (R-HSA:71403, 9 proteins) as response. Also, the cell line up-regulates its transferrin endocytosis and recycling (R-HSA:917977, 5 proteins) while the impact on the signal transduction pathways are represented by i. downregulation of signaling by receptor tyrosine kinases pathway (R-HSA:9006934, 72 proteins) and associated pathways: downregulation of signaling by ERBB4 (R-HSA:1236394, 7 proteins) and IRS-mediated signaling (R-HSA:112399, 6 proteins); ii. downregulation of signaling by TGFB family members (R-HSA:9006936, 18 proteins). The analysis based on the secreted proteins (Supplementary Figure S19, Table S12) showed that, in response to the NCT-503 treatment, Hs 578T presents an upregulation of degradation of the extracellular matrix pathway (R-HSA:1474228, 61 proteins) and elastic fiber formation (R-HSA:1566948, 13 proteins). Downregulation of immune system related pathways such as DAP12 signaling (R-HSA:2424491, 32 proteins), Interleukin-10 (R-HSA:6783783, 7 proteins) and Interleukin-4 and Interleukin-13 (R-HSA:6785807, 5 proteins) signaling pathways were seen for Hs 578T. Also, signaling by MET pathways seem to be negatively affected by treatment (MET activates PTK2 signaling - R-HSA:8874081, 8 proteins and MET promotes cell motility - R-HSA:8875878, 4 proteins).
